# DNA length tunes the fluidity of DNA-based condensates

**DOI:** 10.1016/j.bpj.2021.02.027

**Published:** 2021-02-26

**Authors:** Fernando Muzzopappa, Maud Hertzog, Fabian Erdel

**Affiliations:** 1 MCD, Center for Integrative Biology (CBI), University of Toulouse, CNRS, Toulouse, France

## Abstract

Living organisms typically store their genomic DNA in a condensed form. Mechanistically, DNA condensation can be driven by macromolecular crowding, multivalent cations, or positively charged proteins. At low DNA concentration, condensation triggers the conformational change of individual DNA molecules into a compacted state, with distinct morphologies. Above a critical DNA concentration, condensation goes along with phase separation into a DNA-dilute and a DNA-dense phase. The latter DNA-dense phase can have different material properties and has been reported to be rather liquid-like or solid-like depending on the characteristics of the DNA and the solvent composition. Here, we systematically assess the influence of DNA length on the properties of the resulting condensates. We show that short DNA molecules with sizes below 1 kb can form dynamic liquid-like assemblies when condensation is triggered by polyethylene glycol and magnesium ions, binding of linker histone H1, or nucleosome reconstitution in combination with linker histone H1. With increasing DNA length, molecules preferentially condense into less dynamic more solid-like assemblies, with phage *λ*-DNA with 48.5 kb forming mostly solid-like assemblies under the conditions assessed here. The transition from liquid-like to solid-like condensates appears to be gradual, with DNA molecules of roughly 1–10 kb forming condensates with intermediate properties. Titration experiments with linker histone H1 suggest that the fluidity of condensates depends on the net number of attractive interactions established by each DNA molecule. We conclude that DNA molecules that are much shorter than a typical human gene are able to undergo liquid-liquid phase separation, whereas longer DNA molecules phase separate by default into rather solid-like condensates. We speculate that the local distribution of condensing factors can modulate the effective length of chromosomal domains in the cell. We anticipate that the link between DNA length and fluidity established here will improve our understanding of biomolecular condensates involving DNA.

## Significance

Liquid-liquid phase separation has been proposed to be the organizing principle behind numerous cellular substructures. Chromosomal DNA is highly abundant in the cell nucleus and is actually the main component of some of them. As DNA itself displays rich phase behavior, the properties of nuclear substructures will be determined by the properties of the enclosed DNA and those of the associated molecules. We show here that the DNA length is a critical determinant of the fluidity of DNA condensates, with short and long DNA preferentially forming liquid-like and solid-like condensates, respectively. Our results help predict the material properties of differently sized genomic domains and to put in vitro experiments with DNA molecules that are much shorter than chromosomes into context.

## Introduction

Genomic DNA inside living organisms is typically compacted and condensed as reviewed previously (1,2). Compaction can be promoted by spatial confinement and by condensing agents that favor attractive DNA-DNA interactions. Examples of naturally occurring condensing agents are polyamines and other multivalent cations, positively charged proteins like histones, or cellular constituents that induce macromolecular crowding. The resulting compaction state of genomic DNA appears to be related to its function. Condensed DNA has been shown to be less efficiently transcribed than decondensed DNA in vitro (3), and changes in chromatin compaction have been found to be correlated with transcriptional activity in eukaryotic cells (4). Accordingly, understanding DNA condensation and the properties of the resulting condensates is important for understanding the regulation of DNA-templated cellular processes.

**Figure 1:**
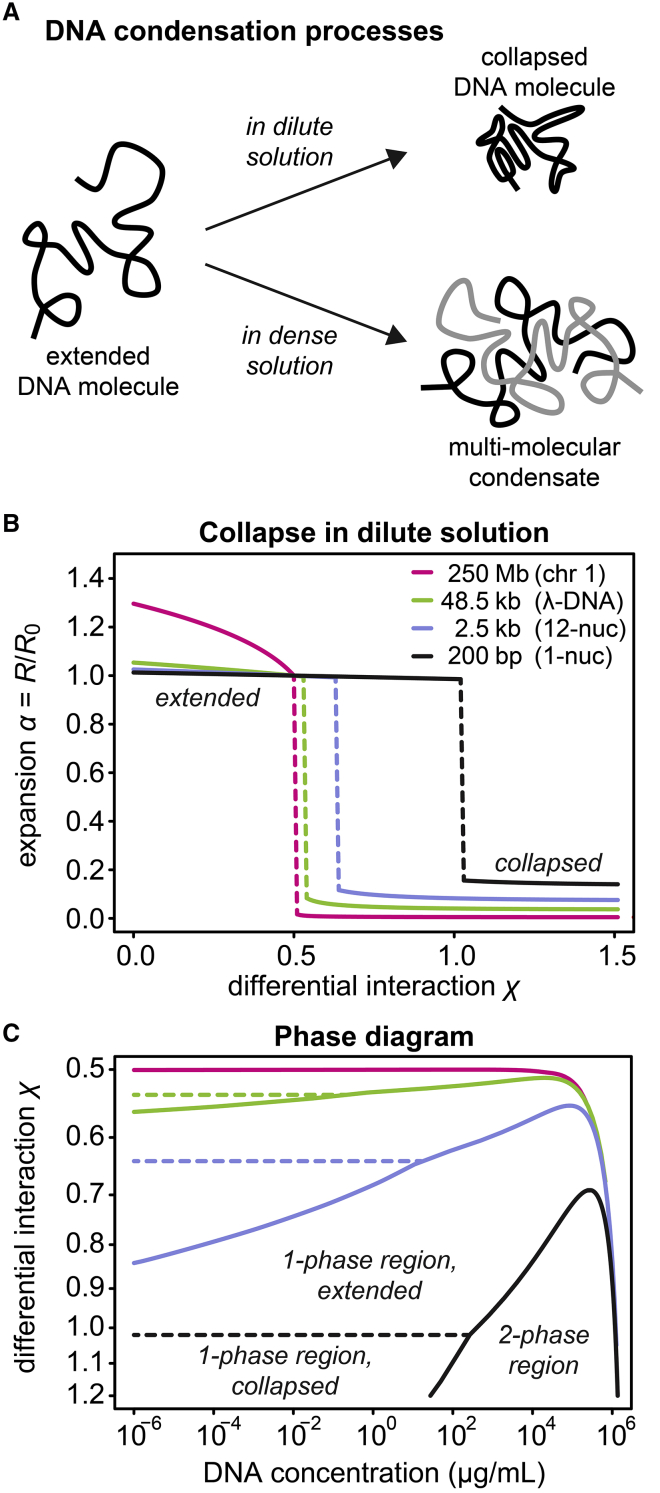
Predicted length dependence of DNA condensation. (*A*) DNA molecules undergo a collapse transition in dilute solution (one-phase region) and form multimolecular condensates in more concentrated solutions (two-phase region). Both processes are predicted to depend on DNA length. (*B*) Length-dependent collapse of DNA molecules in dilute solutions. Longer DNA molecules collapse more easily than shorter DNA molecules. The expansion *α* corresponds to the radius of gyration *R* of the DNA molecules with respect to the radius *R*_0_ in the unperturbed state. The DNA lengths considered here correspond to human chromosome 1 (*magenta*), phage *λ*-DNA (*green*), a 12-mer nucleosomal array (*blue*), and a mononucleosome with adjacent linker DNA (*black*). (*C*) Predicted phase diagrams for differently sized DNA molecules (same lengths and color code as in *B*). Under the same buffer conditions (same *χ*-value), long DNA molecules form multimolecular condensates already at lower concentrations than short DNA molecules. To see this figure in color, go online.

It was recognized several decades ago that DNA molecules can condense in an unfavorable solvent environment, i.e., if DNA-DNA interactions are more favorable than DNA-solvent interactions (5). As depicted in Fig. 1 *A*, condensing DNA molecules in a dilute solution can undergo a conformational change from a relaxed into a compacted state (6,7). In this scenario, DNA molecules remain mostly separated from each other or form small assemblies consisting of only a few DNA molecules. In more concentrated solutions, larger numbers of condensing DNA molecules can associate with one another (7), resulting in multimolecular condensates with different material properties. Because concentrated polymer solutions are typically viscoelastic (8), which means that they share similarities with both viscous liquids and elastic solids, DNA condensates can be expected to fall in the category of viscoelastic materials. Depending on the characteristics of the DNA and the condensing agents in the solvent, condensates have been reported to be rather liquid-like or solid-like. Examples of experimentally observed DNA condensates are the toroidal or rod-like assemblies formed by DNA molecules in the presence of multivalent cations (6,9); the DNA gel particles obtained in concentrated protein solutions or in the presence of a cationic surfactant (10); the liquid crystalline condensates formed by mononucleosomes in crowded solutions (11); the condensates formed by 12-mer nucleosomal arrays in the presence of divalent cations and/or chromatin-associated proteins, which have been reported to behave rather liquid-like (12,13) or solid-like (14) depending on the solvent composition; and the rather solid-like aggregates formed by longer nucleosomal templates under conditions of anionic protein crowding (15).

The DNA molecules used to study condensates in vitro are typically much shorter than the megabase-sized chromosomes present in mammalian cells or the topologically associating domains with sizes of hundreds of kilobases into which chromosomes can be subdivided. Although toroidal assemblies and solid-like aggregates have also been observed for relatively long DNA molecules such as T4 phage DNA with a size of ∼170 kb (15,16), liquid-like condensates have mostly been observed for much shorter DNA molecules, e.g., (11, 12, 13,17, 18, 19). Although it has long been known that DNA length has an influence on the solubility and condensation behavior of DNA, e.g., (20,21), it is largely unclear how DNA length affects the material properties and in particular the fluidity of the resulting condensates in the presence of different condensing agents, making it difficult to compare previous in vitro experiments using differently sized DNA molecules to each other and to the situation in the cell.

To address this issue, we set out to study DNA condensates under different conditions in dependence of DNA length. We used full-length phage *λ*-DNA (*λ*-DNA) and fragments of it to form condensates via polymer- and salt-induced (PSI) condensation (22) by polyethylene glycol (PEG) and magnesium ions, via addition of linker histone H1 in a near-physiological buffer, or via nucleosome reconstitution in combination with addition of H1. Linker histone H1 is an abundant chromatin-associated protein that is involved in the regulation of chromatin structure and that has been shown to promote condensation of DNA and nucleosomal arrays in the test tube (12,18,23,24). We titrated the differently sized DNA species to obtain phase diagrams for PSI and H1-induced condensation, and we investigated the morphology and dynamic properties of the resulting condensates for all three conditions. We found that shorter DNA molecules (<1 kb) tend to form liquid-like condensates that are round-shaped and show exchange with the surrounding medium, whereas longer DNA molecules (>10 kb) tend to form solid-like aggregates with irregular shapes and much less exchange. We observed that condensates made of shorter DNA molecules were less resistant to decondensation induced by removal of condensing agents and incubation in shear flow, whereas condensates made of longer DNA molecules were more resistant. This suggests that the length of DNA molecules in condensates can determine their stability toward mechanical forces. Finally, we found that the DNA length-dependent fluidity is similar for the three types of condensation conditions considered here, suggesting that this phenomenon is a fundamental aspect of the phase behavior of DNA.

## Materials and methods

### Theoretical DNA length dependence of phase diagrams

The total free energy of a DNA solution according to the Flory-Huggins theory and its extension by Post and Zimm (7,25) is given by

$$\begin{array}{l} \frac{\Delta G}{kT}=n_{\text{solvent}}\text{ln}\phi_{\text{solvent}}+n_{\text{DNA}}\text{ln}\phi_{\text{DNA}}+\chi n_{\text{solvent}}\phi_{\text{DNA}}+n_{\text{DNA}}\left[N\left(\left(\chi -1\right)+\frac{B_{2}\omega }{2^{3/2}\alpha^{3}}+\frac{B_{3}\omega^{2}}{2\times 3^{5/2}\alpha^{6}}\right)+\frac{3}{2}\left(\alpha^{2}-1\right)-\text{ln }\alpha^{3}\right]=\frac{V}{V_{\text{s}}}\{\left(1-\phi_{\text{DNA}}\right)\text{ln}\left(1-\phi_{\text{DNA}}\right)+\frac{\phi_{\text{DNA}}}{N}\text{ln}\phi_{\text{DNA}}+\chi \phi_{\text{DNA}}\left(1-\phi_{\text{DNA}}\right)\\ +\frac{\phi_{\text{DNA}}}{N}\left[N\left(\left(\chi -1\right)+\frac{B_{2}\omega }{2^{3/2}\alpha^{3}}+\frac{B_{3}\omega^{2}}{2\times 3^{5/2}\alpha^{6}}\right)+\frac{3}{2}\left(\alpha^{2}-1\right)-\text{ln }\alpha^{3}\right]\}. \end{array}$$

The first three terms correspond to the “external” free energy of mixing, reflecting the arrangement of solvent and “whole” DNA molecules. The remaining terms (square brackets) correspond to the “internal” free energy of mixing of the DNA, describing the arrangement of individual segments within DNA molecules. In the expression above, *n*_DNA_ and *n*_solvent_ are the numbers of DNA and solvent molecules, respectively; *ϕ*_DNA_ and *ϕ*_solvent_ = 1 − *ϕ*_DNA_ are the volume fractions of DNA and solvent, respectively; *V*_s_/*V* is the ratio between the molecular volume of the solvent and the total volume; and *N* is the ratio between the molecular volume of the DNA and that of the solvent, i.e., *N* is proportional to DNA length. *χ* is the interaction parameter that reflects the preference of DNA-DNA interactions over DNA-solvent interactions, and *α* is the expansion parameter that corresponds to the radius of gyration of a DNA molecule with respect to its unperturbed radius of gyration in a *θ*-solvent (at *χ* = 0.5). *B*_2_ and *B*_3_ represent the second and third virial coefficients in the expansion of the osmotic pressure, which are included to account for intersegmental contacts in the collapsed state (5). *ω* is a measure for the flexibility of DNA molecules. The quantities *B*_2_, *B*_3_, and *ω* are defined as

$$\begin{array}{l} B_{2}=\frac{1}{2}-\chi \\ B_{3}=1+12\frac{\chi^{2}}{q}-16\frac{\chi^{3}}{q^{2}}\\ \omega =\;{\left(\frac{9}{\pi <h_{0}^{2}>}\right)}^{3/2}\frac{M_{\text{DNA}}N_{\text{A}}}{\rho_{\text{DNA}}} \end{array}$$

with *q* being the lattice coordination number, $<h_{0}^{2}>$ being the squared unperturbed end-to-end length of a DNA molecule, *M*_DNA_ being the molecular weight of a DNA molecule, *ρ*_DNA_ being the density of DNA, and *N*_A_ being the Avogadro constant. These expressions are based on the description of DNA molecules as Gaussian chains (5,7), which is not the most accurate choice but should capture the general length-dependent phase behavior of DNA, especially for DNA molecules whose length is well-above the persistence length of DNA. Following previous work (5,7), we used the following values for our calculations: *q* = 10, $\rho_{\text{DNA}}=1.8\;\text{g}/{\text{cm}}^{3}$, $\left\langle h_{0}^{2}\right\rangle ={\left[4\times 10^{-15}M_{DNA}N_{A}\right]}^{8/7}cm^{2}/g^{8/7}$, and $N=M_{\text{DNA}}N_{\text{A}}/4000\;{\text{g}}^{-1}$. To determine the expansion *α* of DNA molecules in dilute solution (Fig. 1 *B*), the *α*-value corresponding to the global minimum of the free energy *ΔG* above was determined for different combinations of DNA lengths and interaction parameters *χ*. To find the global minimum of *ΔG*, local extrema were determined by calculating the derivative of *ΔG* with respect to *α* and equating it to zero, yielding the following equation

$$3\alpha^{8}-3\alpha^{6}-N\left(\frac{3B_{2}\omega \alpha^{3}}{2^{3/2}}+\frac{B_{3}\omega^{2}}{3^{3/2}}\right)=0.$$

The free energy *ΔG* was then evaluated at the local extrema and the boundaries of the considered interval (0 ≤ *α* ≤ 1.5), and the smallest value was taken as the global minimum. To determine the phase diagrams in Fig. 1 *C*, volume fractions *ϕ*_DNA, 1/2_ that give the same chemical potentials *μ* for DNA and solvent were determined. The chemical potentials are obtained by differentiating the free energy above with respect to *n*_solvent_ and *n*_DNA_ (7), yielding

$$\begin{array}{l} \frac{\mu_{\text{DNA}}-\mu_{\text{DNA}}^{0}}{kT}=\ln\;\phi_{\text{DNA}}-\left(N-1\right)\left(1-\phi_{\text{DNA}}\right)+\chi N{\left(1-\phi_{\text{DNA}}\right)}^{2}+N\left(\left(\chi -1\right)+\frac{B_{2}\omega }{2^{3/2}\alpha^{3}}+\frac{B_{3}\omega^{2}}{2\times 3^{5/2}\alpha^{6}}\right)+\frac{3}{2}\left(\alpha^{2}-1\right)-\ln\;\alpha^{3}\\ \frac{\mu_{\text{solvent}}-\mu_{\text{solvent}}^{0}}{kT}=\ln\left(1-\phi_{\text{DNA}}\right)+\left(1-1/N\right)\phi_{\text{DNA}}+\chi \phi_{\text{DNA}}^{2}. \end{array}$$

Here, $\mu_{\text{DNA}}^{0}$ and $\mu_{\text{solvent}}^{0}$ are the chemical potentials of the pure phases. For the dilute phase, the expansion parameter *α* was calculated as described above. For the dense phase, the unperturbed expansion parameter *α* (for a *θ*-solvent with *χ* = 0.5) was used because DNA molecules are expected to establish sufficient intermolecular interactions with neighboring DNA molecules so that the entropically unfavorable collapse, which increases the number of intramolecular DNA-DNA interactions in dilute solution, does not occur. Finally, the resulting volume fractions *ϕ*_DNA, 1/2_ were converted into concentrations *c*_DNA, 1/2_ by multiplying them with the density *ρ*_DNA_ given above.

### DNA substrates and labeling

Wild-type *λ*-DNA (N3011) was purchased from New England Biolabs (NEB, Ipswich, MA). To label *λ*-DNA for confocal microscopy experiments, 50 *μ*g of *λ*-DNA was premixed at 65°C with a 300-fold excess of Cy3-labeled oligonucleotides matching the *cos* sites (Lambda_R and Lambda_L, Table S1) and was then ligated with T4 ligase (EL0011; Thermo Fisher Scientific, Waltham, MA) for 2 h at 37°C. Labeled DNA was purified and separated from free Cy3-oligonucleotides through precipitation by addition of 10% PEG 8000 and 10 mM MgCl_2_. To generate *λ*-DNA fragments of different sizes (Fig. S1, *A*–*C*), 1.5 *μ*g of labeled *λ*-DNA was incubated at 37°C for 2 h with 1) AfeI (NEB, R0652); 2) BamHI (NEB, R3136) and KpnI (NEB, R0142); 3) AatII (NEB, R0117), BamHI (NEB, R3136), and KpnI (NEB, R0142); or 4) AciI (NEB, R0551), *Hin*dIII (NEB, R0104), and DraI (NEB, R0129). The 1 kb and 5 kb fragments were generated by PCR using the oligonucleotides LambdaFW and Lambda1_rv/Lambda5_rv or LambdaRV and Lambda1_fw/Lambda5_fw, yielding fragments with the respective length from the AT-rich or the CG-rich part of *λ*-DNA. For both lengths (1 kb and 5 kb), the resulting AT- or CG-rich fragments were mixed at equimolar ratio. The size distribution obtained in each reaction was determined by DNA gel electrophoresis in 0.8% agarose (05077; Sigma-Aldrich, St. Louis, MO). To obtain 12 base pair (bp) double stranded DNA fragments, Cy3-labeled oligonucleotides matching the *cos* sites were annealed to each other. For total internal reflection fluorescence (TIRF) experiments, the *cos* overhangs of *λ*-DNA were filled with CTP-biotin (Thermo Fisher Scientific) and aminoallyl-UTP (Thermo Fisher Scientific) using DNA Polymerase I Klenow (NEB). Aminoallyl-UTP was subsequently labeled using Atto 488 NHS (succinimidyl ester; Sigma-Aldrich) or Atto 565 NHS (Sigma-Aldrich).

### Phase separation assay, fluorescence microscopy, and fluorescence recovery after photobleaching

To induce phase separation, DNA was incubated in PSI condensation buffer (25 mM Tris⋅OAc (pH 7.5), 0.1 mM EDTA, 5 mM dithiothreitol (DTT), 300 mM KOAc, 2 mM Mg[OAc]_2_, 50 mM MgCl_2_, and 7.5% PEG 8000) or H1 buffer (25 mM Tris⋅OAc (pH 7.5), 0.1 mM EDTA, 5 mM DTT, 300 mM KOAc, 2 mM Mg[OAc]_2_) for 30 min at room temperature. For H1-induced condensation, human linker histone H1^0^ (NEB, M2501) was added at an H1:DNA mass ratio of 1:6, corresponding to roughly one H1 molecule per 200 bp of DNA. Subsequently, samples were gently mixed and pipetted on a microscopy slide, which was precoated with PEG 8000. All experiments were carried out at room temperature within 20 min after pipetting.

Confocal fluorescence microscopy and fluorescence recovery after photobleaching (FRAP) experiments were performed on a Zeiss LSM 710 confocal light scanning microscope (Carl Zeiss, Oberkochen, Germany), equipped with a 63×/NA 1.2 oil immersion objective. Image analysis was conducted in R using the EBImage package (25), and the aspect ratio was calculated as

$$\text{aspect}\;\text{ratio}=\frac{R_{\text{max}}-R_{\text{min}}}{R_{\text{max}}+R_{\text{min}}}.$$

Here, *R*_max_ and *R*_min_ are the maximum and minimum radius of the segmented structure, respectively. This expression vanishes for a circle and approaches unity for an elongated rod. For FRAP experiments, images were acquired at 128 × 512 pixels at a scan speed corresponding to 200 ms per image. Typically, 300 images were acquired over 5 min (with an interval of 800 ms between subsequent images). The FRAP analysis was conducted in R, using the following equation to fit normalized curves:

FRAP(t)=a×(1−exp(−kt))

Here, *k* is the apparent exchange rate and *a* is the apparent mobile fraction. The half time of recovery was calculated according to $t_{1/2}=-\text{ln}\left(0.5\right)/k$. Because condensates residing on the surface of the cover glass were used for the experiments, FRAP curves might contain a contribution from DNA molecules that did not exchange because of interactions with the glass surface. To identify the different types of DNA condensates in Fig. 6, hierarchical clustering was performed using the R package pheatmap. Mobile fractions and aspect ratios were clustered into three groups using Euclidean distances and the complete-linkage clustering method.

For in situ digestions of DNA condensates, 5 *μ*L of Cy3-labeled *λ*-DNA (digested to 17 kb) were placed on a microscopy slide, and 1 *μ*L of micrococcal nuclease (NEB, M0247) was added. Images were acquired for 20 min after pipetting, with an interval of 1 s between images.

### H1 titration assay

To address the effect of the H1:DNA stoichiometry on the material properties of DNA condensates, we induced DNA condensation of 36 kb *λ*-DNA fragments by adding H1 at different concentrations (0.06, 0.3, 0.61, 3.06, and 6.12 μM). These different H1:DNA ratio yield in the following stoichiometries: 0.2 H1/200 bp DNA, 1 H1/200 bp DNA, 2 H1/200 bp DNA, 10 H1/200 bp DNA, and 20 H1/200 bp DNA. After 30 min of incubation at room temperature, the samples were placed on a microscope slide, and the condensates were analyzed by FRAP as described above.

### Reconstitution of chromatinized DNA and induction of phase separation

Reconstitution of chromatinized DNA was carried out using the Cy3-labeled *λ*-DNA of various lengths described above. Briefly, 0.5 *μ*g of DNA was mixed with equimolar quantities of histones H2A/H2B (NEB, M2508S), H3.1 (NEB, M2504S), and H4 (NEB, M2508S) relative to the number of nucleosome binding sites (defined as 200 bp, including linker DNA) in reconstitution buffer (10 mM Tris-HCl (pH 7.6), 1 mM EDTA, 0.1% Igepal, 3 M NaCl, and 10% glycerol). The samples were moved to 6000 Da MWCO dialysis chambers and equilibrated with 500 mL of high-salt buffer (10 mM Tris-HCl (pH 7.6), 1 mM EDTA, 2 M KCl, 2 mM DTT). First, the salt concentration was lowered by continuous addition of 500 mL of low-salt buffer (10 mM Tris-HCl (pH 7.6), 1 mM EDTA, 200 mM KCl, 2 mM DTT) using a peristaltic pump at 0.8 mL/min at 4°C. Subsequently, the volume was reduced to 100 mL, and the salt concentration was further reduced by continuous addition of 1 L of no-salt buffer (10 mM Tris-HCl (pH 7.6), 1 mM EDTA, 10% glycerol, 2 mM DTT) at 0.8 mL/min at 4°C.

To induce phase separation, chromatinized DNA was diluted in dilution buffer (25 mM Tris-OAc (pH 7.5), 2.5 mM DTT, 0.1 mM EDTA, 0.1 mg/mL bovine serum albumin (BSA), 5% glycerol) to a final nucleosome concentration of 300 nM. Then, an equal volume of H1 (120 nM) in H1 buffer was added. The samples were mixed and incubated at room temperature for 30 min before visualization by confocal microscopy.

To confirm the presence of histones in the condensates, 2 *μ*L of chromatinized DNA was mixed with 1 *μ*L of anti-histone H4-Alexa Fluor 647 (ab207075; Abcam, Cambridge, UK), diluted 1:400, and 1 *μ*L of anti-histone H2A/H2B-Atto 488 (tba488-100; Chromotek, Planegg-Martinsried, Germany), diluted 1:400 (Fig. S1 *D*).

### Single-molecule TIRF microscopy

DNA condensates prepared as described above were injected into a flow cell and tethered to the surface of a coverslip via biotin-streptavidin interactions. Flow cells (4 × 0.4 × 0.07 mm) were assembled using a glass slide, a coverslip, and double-sided tape (3M Adhesive Transfer Tape; St. Paul, MN). Ports were drilled into the glass slide, and flow was controlled using a pressure-driven pump (Fluigent, North Chelmsford, MA). The surface of the coverslip was functionalized by injecting filtrated TN buffer (25 mM Tris⋅Cl (pH 7.5), 50 mM NaOAc) containing 2 mg/mL biotin-BSA (Pierce, Thermo Fisher Scientific) and incubating for 10 min. Subsequently, the surface was blocked with injection of TN buffer containing 0.2% BSA for 10 min, equilibrated with 0.2 mg/mL streptavidin (Promega, Madison, WI) for 10 min, and then blocked again with 1.5 mg/mL Roche Blocking Reagent (Roche, Basel, Switzerland) for 30 min. For imaging, DNA condensates were incubated in the flow cell in the absence of flow for ∼20 min. Surface-anchored DNA condensates were then visualized by a TIRF microscope (Nikon, Tokyo, Japan) using Atto 488 (A488), and Atto 565 (A565) fluorescence. Next, PSI condensation buffer adapted for TIRF (25 mM Tris⋅OAc (pH 7.5), 0.05 mM EDTA, 2.5 mM DTT, 7.5% PEG 8000, 60 mM Mg[OAc]_2_) or decondensation buffer (PSI buffer without PEG and with only 1 mM Mg[OAc]_2_) were continuously injected using a differential pressure of 25 mbar, corresponding to flow rates of 30 *μ*L/min for PSI condensation buffer and 80 *μ*L/min for decondensation buffer. Fluorescence intensities of condensates containing A488- and A565-labeled DNA were recorded over time and quantified. Image analysis was conducted in R using the EBImage package (26). In brief, images were registered, DNA condensates were segmented, the maximum fluorescence intensity among the pixels corresponding to each condensate was quantified at each time step, and the mean background intensity in a ring around each condensate was subtracted. Only condensates that were visible in both color channels were kept for the subsequent analyses. The boxplots in Fig. 4 *C* show the intensities of DNA condensates in the A488 channel divided by the intensity of a single A488 dye and by the fraction of A488-labeled DNA molecules in the mix. The intensity of single A488 dyes was obtained in a control experiment in which A488-labeled dsOligos were anchored to the surface without inducing condensation. The intensity curves shown in Fig. 4, *D* and *E* correspond to the mean of the signals across all (double-labeled) condensates along with the standard error of the mean (SEM).

## Results

### DNA condensation depends on DNA length

To gauge the expected influence of DNA length on the condensation process from first principles, we briefly recall the Flory-Huggins theory and its extension by Post and Zimm (5,7,25). Both the collapse of DNA molecules in dilute solution and the formation of multimolecular condensates in more concentrated solutions (Fig. 1 *A*) are predicted to be length dependent. The underlying reason is that both the “internal” free energy that accounts for the conformation of individual DNA molecules and the “external” free energy of mixing between DNA and solvent molecules at a given mass concentration depend on DNA length (see Materials and methods). Fig. 1 *B* shows the predicted compaction of DNA molecules with different lengths under varying solvent conditions. Here, the expansion *α* corresponds to the radius of gyration of the DNA molecule compared to the situation in the unperturbed state, and the solvent condition is characterized by the interaction parameter *χ*, which reflects the preference of DNA-DNA interactions over DNA-solvent interactions. Longer DNA molecules collapse more easily than shorter DNA molecules, which is evident from the poorer solvents (larger *χ*-values, stronger DNA-DNA interactions) that are required to induce collapse of shorter DNA molecules (Fig. 1 *B*). In solvents that are poor enough to induce the collapse of both short and long DNA molecules (*χ* >> 1), the compaction degree of long DNA molecules is expected to be higher than for short DNA molecules. Fig. 1 *C* shows the predicted phase diagrams for DNA molecules with the lengths considered in Fig. 1 *B*, including those of mammalian chromosomes and of popular templates used in in vitro studies. The dashed lines mark the collapse transition in dilute solutions, and the solid lines reflect the DNA concentrations in the two phases, i.e., multimolecular condensates and surrounding dilute phase, which are formed at higher DNA concentrations. For example, in a solvent with *χ* = 0.7, phase separation of DNA molecules with a length of 2.5 kb (*blue line*) and 200 bp (*black line*) is predicted to occur at ∼0.3 *μ*g/mL and ∼160 mg/mL, respectively. Accordingly, the critical concentration that defines the onset of phase separation is expected to depend strongly on DNA length.

Although the theory above readily predicts the DNA length-dependent compaction degree and the critical concentrations, it does not provide a straightforward prediction of the material properties of the condensates. Accordingly, without additional assumptions about the specifics of the DNA-DNA interactions and the conformation and entanglement of DNA molecules in the condensates, it is unclear under which conditions the condensation process might be understood as liquid-liquid phase separation, gelation, or aggregation, which produce molecular assemblies with distinct morphology, stability, and internal dynamics.

### DNA length determines the morphology of DNA condensates

To experimentally assess the impact of DNA length on the condensation process, we studied DNA condensation of differently sized DNA molecules under three distinct sets of conditions. To this end, we digested *λ*-DNA with different combinations of restriction enzymes to obtain samples with different DNA length distributions (see Materials and methods and Fig. S1). We also produced *λ*-DNA fragments by PCR to obtain DNA lengths that were not readily obtained by digestion. We then induced DNA condensation by addition of PEG and Mg^2+^ (hereafter referred to as PSI condensation), by addition of linker histone H1 (hereafter referred to as H1 condensation), or by nucleosome reconstitution and addition of H1 (hereafter referred to as chromatinization). Whereas PSI condensation involves salt-dependent charge neutralization and crowding-induced DNA-DNA attraction, H1 condensation and chromatinization are based on the capability of positively charged histones to bind and bridge DNA molecules.

**Figure 2:**
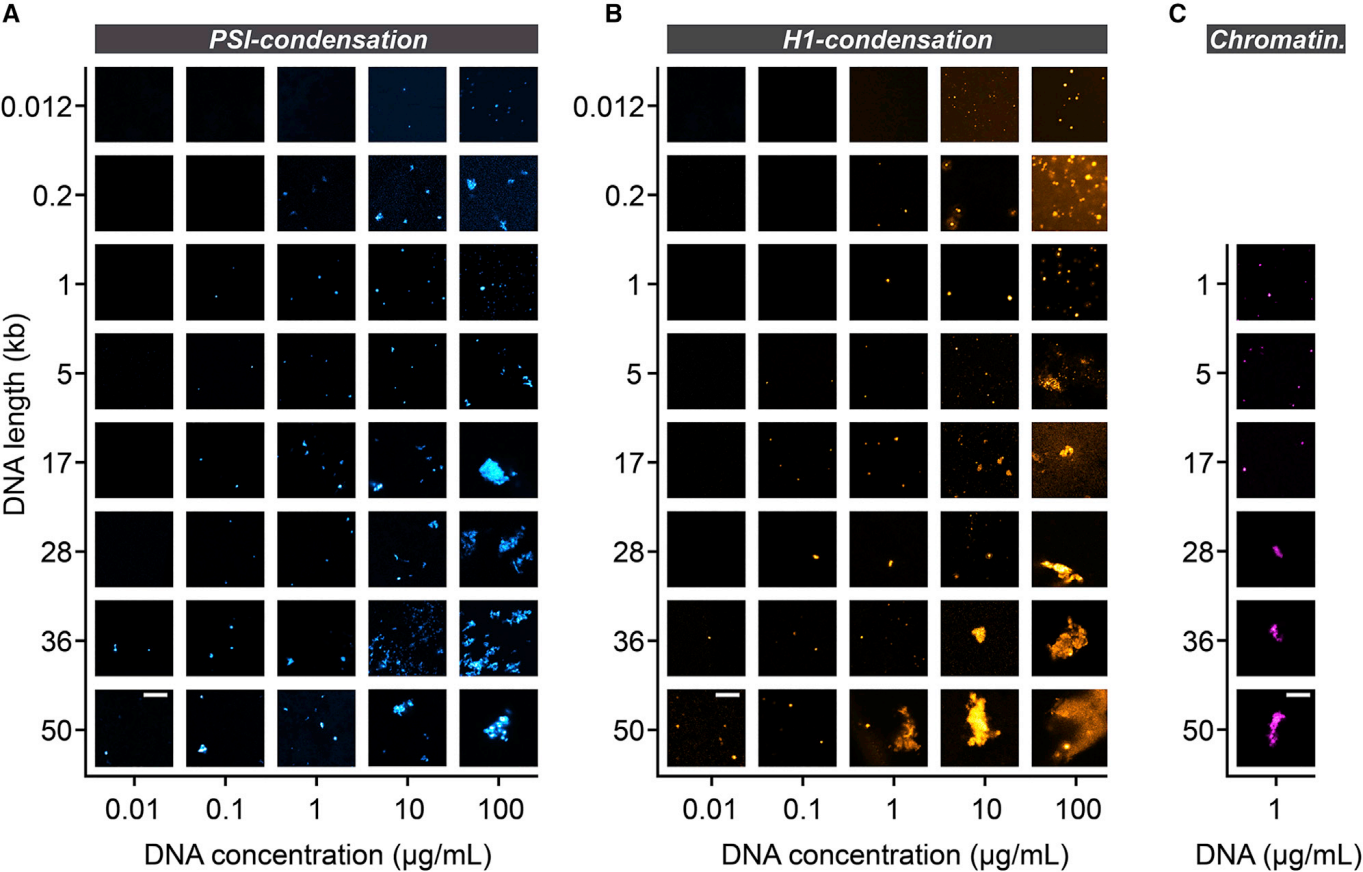
Morphology of DNA condensates. (*A*) DNA condensates formed in the presence of polyethylene glycol and magnesium ions (PSI condensation). All images are equally scaled. Scale bar in the bottom left image, 10 *μ*m. (*B*) DNA condensates formed in the presence of linker histone H1. All images are equally scaled. Scale bar in the bottom left image, 10 *μ*m. (*C*) DNA condensates formed with reconstituted chromatin in the presence of linker histone H1. All images are equally scaled. Scale bar in the bottom image, 10 *μ*m. To see this figure in color, go online.

For the case of PSI condensation, *λ*-DNA with a length of 48.5 kb formed condensates that were visible under the microscope at all DNA concentrations tested here, which ranged from 10 ng/mL to 100 *μ*g/mL (Fig. 2 *A*). Shorter DNA molecules formed visible condensates only at higher DNA concentrations. The morphology of the resulting condensates depended both on the concentration and the length of the DNA. Longer DNA molecules (≥17 kb) formed larger condensates with irregular shapes at high concentrations (∼10–100 *μ*g/mL) and smaller condensates with either round or irregular shapes at lower concentrations (∼10–100 ng/mL). Shorter DNA molecules (≤1 kb) formed condensates at high concentration that resembled those observed for longer DNA molecules at lower concentrations. To systematically quantify the morphology of the individual condensates, we calculated their aspect ratio (Fig. S2 *A*; Table S2). This analysis showed that condensates were on average more round-shaped close to the critical concentration and less round-shaped deeper within the two-phase region. The condensate size showed a similar trend as the aspect ratio (Fig. S2 *B*). Because the shape of small condensates might not be properly resolved in our microscopy images, we reassessed the largest condensates we obtained with small DNA molecules (12 bp). Most of these condensates were round-shaped (see example in Fig. S3 *A*), indicating that short DNA molecules can indeed condense into round structures. To further confirm that the morphology of DNA condensates depended on DNA length, we digested *λ*-DNA within irregular condensates in situ. Addition of micrococcal nuclease rapidly transformed irregular condensates into smaller and more round-shaped ones (Fig. S3 *C*), suggesting that condensates can transition into a more liquid-like state when DNA length decreases.

We next induced DNA condensation by addition of linker histone H1 in a near-physiological buffer (Fig. 2 *B*). We observed similar trends as for PSI condensation under these conditions: Longer DNA molecules (≥5 kb) formed irregularly shaped aggregates at high concentrations and smaller more round-shaped ones at lower concentrations, whereas shorter DNA molecules (≤1 kb) tended to form round-shaped condensates. This is also reflected by the aspect ratio (Fig. S2 *A*). In contrast to PSI DNA condensates, H1-induced condensates appeared more round-shaped close to the critical concentration and also formed structures that resembled fusion intermediates (Fig. S3 *B*). Notably, H1 without DNA did not form visible condensates under the conditions used here (Fig. S3 *D*). Next, we assessed the morphology of condensates containing reconstituted chromatin in the presence of linker histone H1 (Fig. 2 *C*; Table S2). Also in this case, longer DNA molecules (≥28 kb) formed irregular assemblies, and shorter DNA molecules (≤17 kb) formed more round-shaped condensates. We did not acquire a phase diagram for this condition but chose a commonly used concentration range (12,14) because nucleosome arrays reconstituted at higher concentrations tended to condense already without addition of H1, which might complicate the analysis if this process is not fully reversible.

### DNA length affects the internal dynamics of DNA condensates

**Figure 3:**
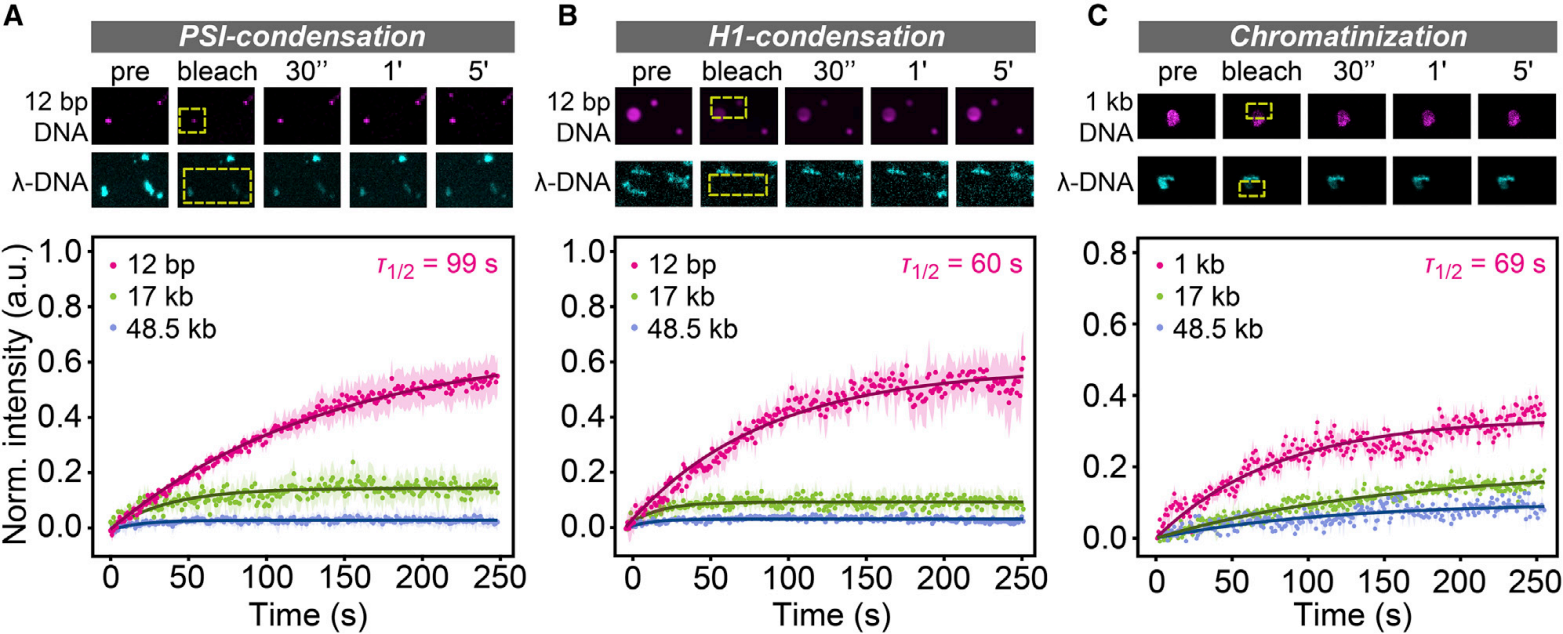
Dynamic properties of DNA condensates. (*A*) Snapshots of PSI DNA condensates during FRAP experiments are shown at the top. The DNA concentration was 100 *μ*g/mL. Yellow rectangles mark the bleach regions. FRAP curves for condensates containing DNA molecules with the indicated lengths are shown below. Error bars represent SEM. (*B*) Same as (*A*) but for H1 condensation. (*C*) Same as (*A*) but for reconstituted chromatin with H1 at 1 *μ*g/mL. To see this figure in color, go online.

To investigate the dynamic properties of the different types of DNA condensates introduced above, we used FRAP. Long DNA molecules within irregularly shaped PSI condensates showed almost no fluorescence recovery after having been bleached (*blue curve* in Fig. 3 *A*; see Table S3 for fit results), indicating that there is no fast exchange of molecules between these condensates and the surrounding dilute phase. Partial photobleaching suggested that the internal dynamics of these DNA condensates are also slow, consistent with a gel- or solid-like state (Fig. S4). Round-shaped condensates fell into two groups with different mobility: whereas longer DNA molecules (≥17 kb) were relatively immobile within such condensates (*green curve* in Fig. 3 *A*), even at low DNA concentrations (Table S3), shorter DNA molecules (≤200 bp) showed significant exchange between condensates and the surrounding dilute phase (*magenta curve* in Fig. 3 *A*, mobile fraction of ∼66% and apparent time constant of ∼99 s). The mobility of short 12 bp DNA in condensates decreased when (unlabeled) full-length *λ*-DNA was added (Fig. S5), indicating that long DNA molecules can reduce the overall fluidity of condensates. Also in the case of H1 condensation, assemblies involving short DNA molecules were more dynamic (*magenta curve* in Fig. 3 *B*, mobile fraction of ∼58% and apparent time constant of ∼60 s) than those containing longer DNA molecules (*green* and *blue curves* in Fig. 3 *B*). However, in contrast to PSI condensation, the dynamics of condensates also depended on the DNA concentration; at low concentrations that were close to the critical concentration, long DNA fragments were slightly more mobile within the condensates (mobile fraction of ∼20–30%) compared with DNA molecules of the same length in condensates formed at higher concentrations (Fig. S6). Finally, condensates formed with reconstituted chromatin showed similar length-dependent dynamics as condensates formed under the other two sets of conditions, with shorter molecules being more mobile (*magenta curve* in Fig. 3 *C*, mobile fraction of ∼40% and apparent time constant of ∼69 s) than longer molecules (*green* and *blue curves* in Fig. 3 *C*).

### DNA length affects the stability of DNA condensates in flow

**Figure 4:**
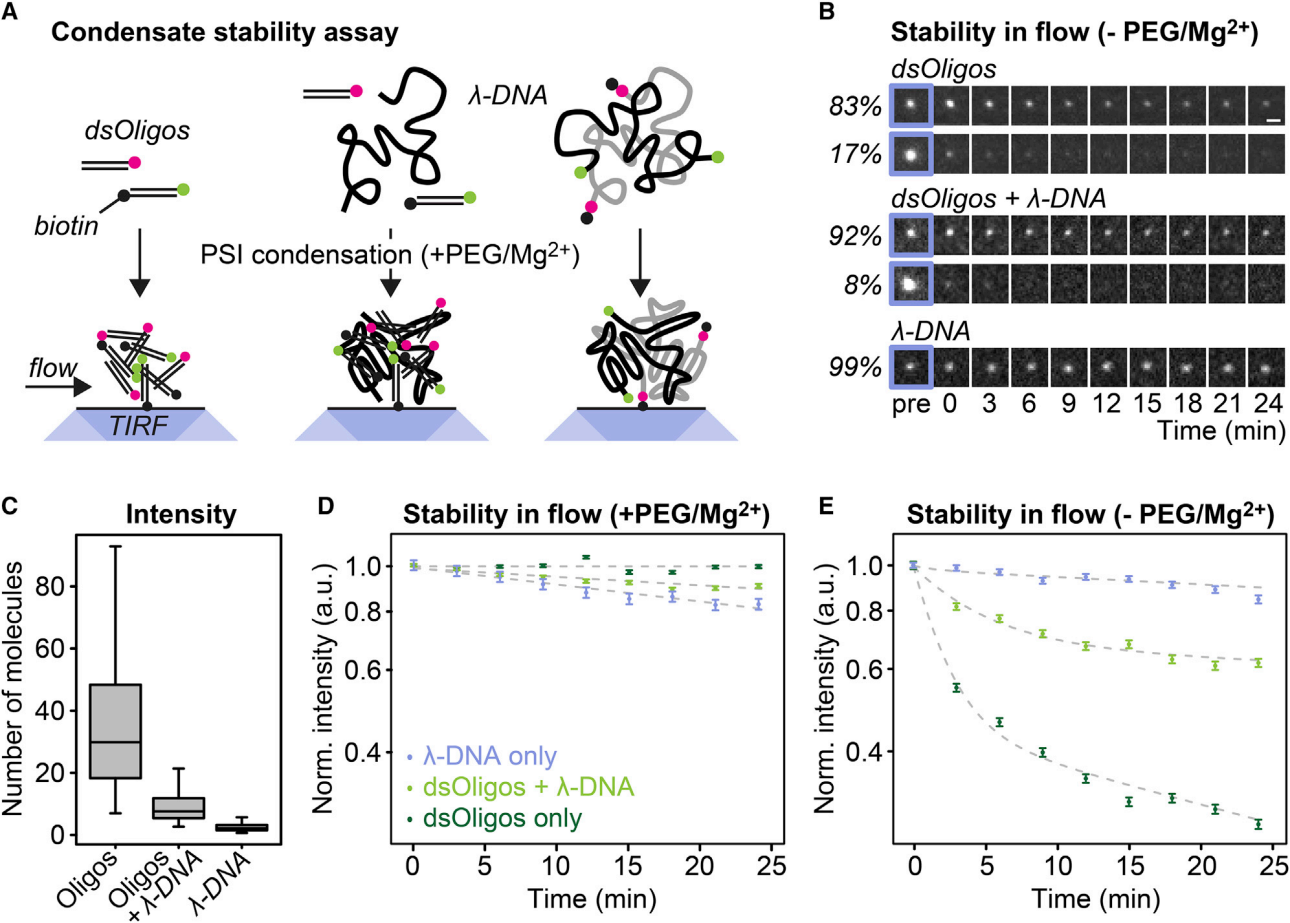
Stability of DNA condensates in flow. (*A*) Scheme of the experimental setup to assess the stability of individual DNA condensates. (*B*) Snapshots of representative DNA condensates challenged with decondensation buffer. All images are equally scaled. Scale bar in the top right image, 1 *μ*m. (*C*) Number of DNA molecules per condensate estimated from their fluorescence intensity. (*D*) Stability of DNA condensates in PSI condensation buffer under flow. The signal in the green channel is shown. Error bars, SEM. (*E*) Same as (*D*) but for decondensation buffer. To see this figure in color, go online.

To test whether the different dynamic properties of condensates observed above are paralleled by a different resistance toward perturbations, we exposed DNA condensates to flow of different buffers and investigated their stability using TIRF microscopy. In particular, we prepared PSI DNA condensates containing either short double-stranded DNA oligomers with a length of 12 bp (dsOligos; Fig. 4 *A*, *left*), a mix of these dsOligos and the same mass of *λ*-DNA (Fig. 4 *A*, *center*), or *λ*-DNA alone (Fig. 4 *A*, *right*). For the first two cases, one half of the dsOligos was labeled with Atto 565 and the other half was labeled with Atto 488 and coupled to biotin (Table S1). For the latter case, one half of the *λ*-DNA was labeled with Atto 488 and the other half was labeled with Atto 565 and coupled to biotin. DNA condensates were attached to the surface of the streptavidin-coated flow chamber and were imaged under flow in different buffers (Fig. 4 *B*). Based on their intensity, we estimated that condensates with short dsOligos contained ∼20–50 DNA molecules, mixed condensates 5–12 DNA molecules, and *λ*-DNA condensates two to four DNA molecules (Fig. 4 *C*; Materials and methods). We exposed DNA condensates to continuous flow of PSI condensation buffer and assessed their stability. Under these conditions, all three types of condensates remained rather stable (Figs. 4 *D* and S7 *A*), showing that they can resist the flow-induced force in the chamber. Next, we exposed DNA condensates to continuous flow in the same buffer without PEG and with less magnesium (Figs. 4 *E* and S7 *B*). Under these conditions, DNA condensates containing short dsOligos disassembled over the course of the experiment, whereas condensates containing only *λ*-DNA remained rather stable. For condensates with dsOligos, we observed two populations with different kinetics: a smaller population that disassembled quickly, with most of the signal having vanished after 3 min, and a larger population that disassembled gradually over the course of >25 min. These two populations might correspond to condensates that are anchored to the surface by different numbers of biotinylated dsOligos. The decay kinetics for condensates containing dsOligos followed a double-exponential decay (Fig. 4 *E*, *dashed lines*), with condensates containing only dsOligos decaying faster than mixed condensates containing dsOligos and *λ*-DNA (Table S4).

These results indicate that attractive interactions among short 12 bp DNA molecules can stabilize condensates for a few minutes under the conditions used here, whereas interactions among longer *λ*-DNA molecules stabilize condensates for at least 25 min. This length-dependent stability is in line with the length-dependent dynamics of DNA condensates observed in the FRAP experiments above, with the rates for the fast component of the decays (Table S4) being very similar to the rates obtained by FRAP (Table S3).

### H1:DNA stoichiometry regulates the dynamics of DNA condensates

**Figure 5:**
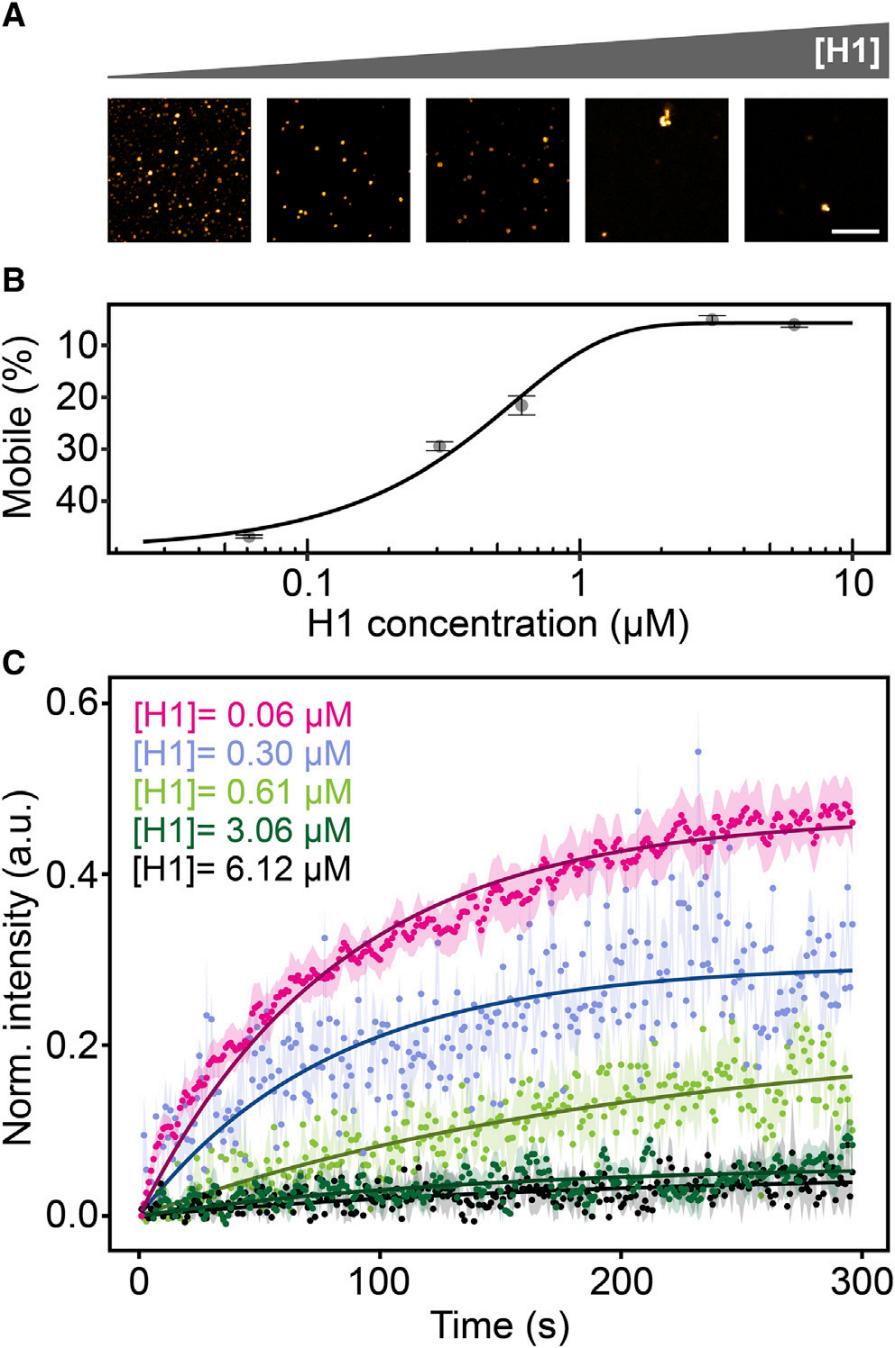
H1-dependent dynamics of DNA condensates. (*A*) Morphology of condensates with 36 kb DNA molecules at different H1:DNA ratios. The different stoichiometries were obtained by adding H1 at different concentrations (from *left* to *right*: 0.06, 0.3, 0.61, 3.06, and 6.12 *μ*M). (*B*) Mobile fraction for condensates with different H1:DNA stoichiometries. (*C*) FRAP curves for condensates with different H1:DNA stoichiometries. Error bars, SEM. To see this figure in color, go online.

Having established that the properties of DNA condensates depend on DNA length, we wondered whether this length dependence might be due to the different number of interaction points among short DNA molecules and among long DNA molecules. According to this hypothesis, long DNA molecules with fewer interaction points would be expected to behave like short DNA molecules. Alternatively, the length dependence might have other reasons, e.g., a different degree of entanglement in condensates with short and long DNA molecules, which would be rather independent of the number of interaction points. To test these ideas, we titrated a solution of DNA molecules with a size of 36 kb with increasing amounts of linker histone H1. We reasoned that the H1 density along the DNA should scale with the H1:DNA stoichiometry, so that the number of H1-dependent bridging points changes accordingly. With increasing H1:DNA stoichiometry, the shape of condensates changed from round to irregular (Fig. 5 *A*) and the mobile fraction in FRAP experiments decreased (Fig. 5, *B* and *C*). Thus, the H1:DNA ratio critically determines the fluidity of the condensates.

These results suggest that the DNA length-dependent material properties of DNA condensates described above arise from the different numbers of attractive interactions that are established among DNA molecules of different length. Accordingly, the physical length of a DNA molecule in combination with the density of condensing agents seem to define an “effective length,” which governs the behavior of the molecule. If condensing agents are site-specifically bound along a DNA molecule, e.g., a chromosome, it is conceivable that regions with lower and higher densities of condensing agents will show similarities with shorter and longer DNA molecules, respectively.

### Phase diagrams link DNA length with the critical concentration, morphology, and dynamic properties of condensates

**Figure 6:**
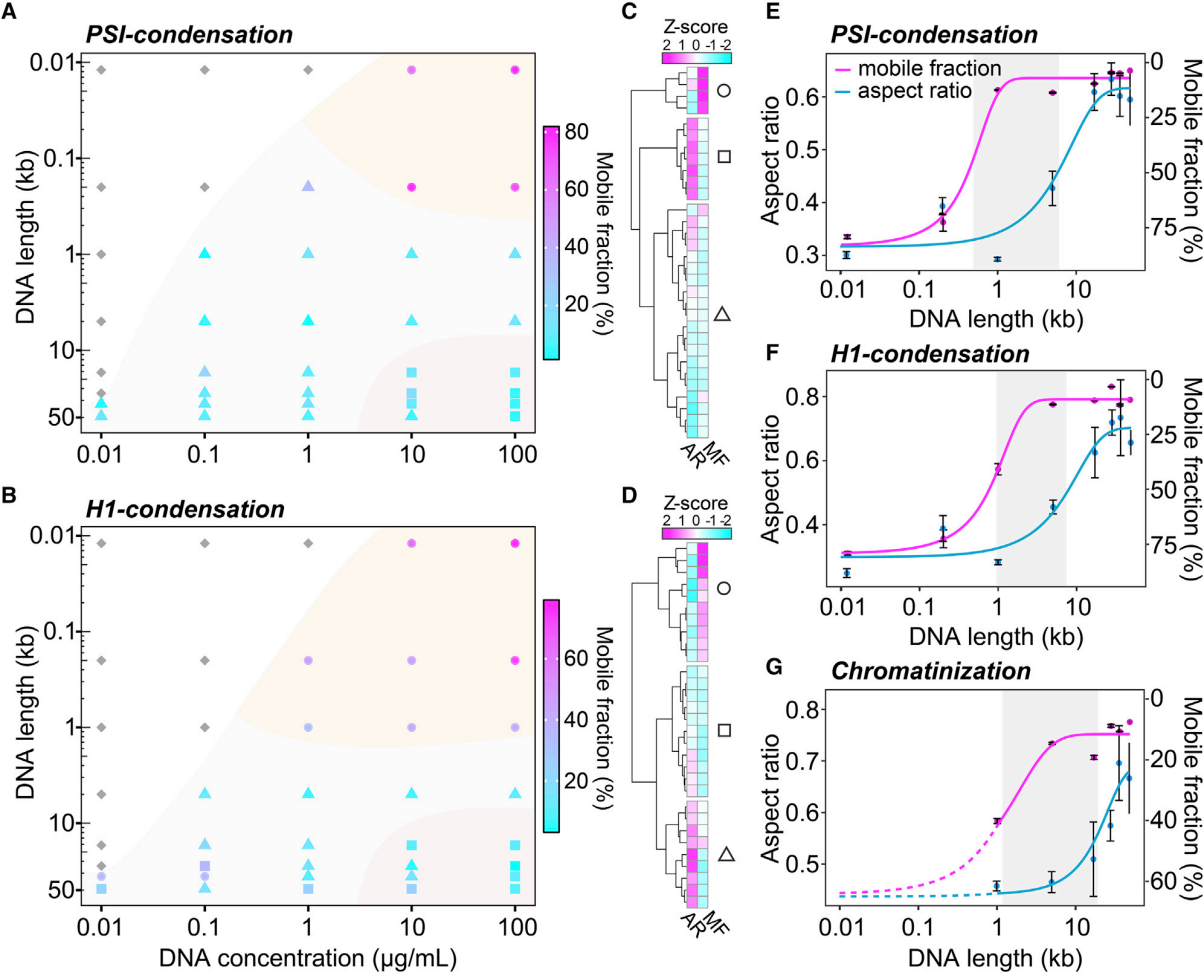
For a Figure360 author presentation of this figure, see https://doi.org/10.1016/j.bpj.2021.02.027. Phase diagrams for DNA condensation induced under different conditions. (*A*) Phase diagram for DNA condensation induced by PEG and magnesium ions. The mobile fraction of the respective condensate is color coded (see *color bar to the right*). Clusters of condensates based on the aspect ratio and the mobile fraction are denoted by the different shapes: dynamic round-shaped assemblies (*circles*), rather static round-shaped assemblies (*triangles*), and static irregularly shaped assemblies (*squares*). Gray diamonds denote the absence of visible condensates. (*B*) Same as (*A*) but for DNA condensates induced by addition of linker histone H1. (*C*) Clustered heatmap of normalized aspect ratios (AR) and mobile fractions (MF) for the DNA condensates in (*A*). (*D*) Same as (*C*) but for DNA condensates in (*B*). (*E*) Aspect ratio (*cyan*) and mobile fraction (*magenta*) as functions of the DNA length in PSI DNA condensates at 100 *μ*g/mL. Gray regions are demarcated by the inflection points of the two curves. Error bars, SEM. (*F*) Same as (*E*) but for H1-induced DNA condensates. (*G*) Same as (*E*) but for reconstituted chromatin with H1 at 1 *μ*g/mL. To see this figure in color, go online.

To systematically integrate the influence of DNA length and DNA concentration on the morphology and dynamics of DNA condensates, we generated phase diagrams for both PSI condensation and H1 condensation (Fig. 6, *A* and *B*). These diagrams show that the critical concentration decreases with increasing DNA length, confirming that long DNA molecules condense more easily than short ones. We observed a similar length dependence when comparing PSI condensation to H1 condensation, suggesting that this relationship might be relatively independent of the condensation mechanism. We clustered the observed condensates according to their aspect ratio and mobile fraction (Fig. 6, *C* and *D*), and defined three groups corresponding to dynamic round-shaped assemblies (*circles*), rather static round-shaped assemblies (*triangles*), and static irregular assemblies (*squares*). These different types of condensates occur in distinct regions of the phase diagrams, which we call liquid-like (region I, *circles*), intermediate (region II, mostly *triangles*), and solid-like (region III, mostly *squares*). The shape of regions I/II differs between PSI condensation and H1 condensation (compare Fig. 6 *A* vs. *B*). While dynamic condensates are only found for short DNA molecules in the case of PSI condensation, dynamic condensates are also found for long DNA molecules at low DNA concentrations for H1 condensation. This difference might be due to the fact that the amount of DNA-bound H1 decreases when concentrations drop below the dissociation constant for H1-DNA interactions, whereas PSI condensation driven by PEG and magnesium might not involve this type of concentration-dependent bridging interaction. When plotting the aspect ratio and the mobile fraction for the highest DNA concentration in the phase diagram, it becomes apparent that the properties of DNA condensates gradually change with DNA length (Fig. 6, *E* and *F*). For all three conditions used here, the transition from dynamic round-shaped to static irregular assemblies occurred between 1 and 10 kb. With increasing DNA length, condensates first changed their dynamics (*magenta line* in Fig. 6, *E* and *F*) and then changed their morphology (*cyan lines* in Fig. 6, *E* and *F*), giving rise to an intermediate regime with solid-like round-shaped condensates. These condensates might correspond to aged liquid droplets whose shape was initially influenced by interfacial tension before they converted into the more solid-like structures we observed here. Following this reasoning, condensates containing long DNA molecules such as *λ*-DNA with 48.5 kb would not qualify as aged liquid droplets because they exhibit irregular morphologies under all condensation conditions assessed here.

## Discussion

The formation of biomolecular condensates by phase separation has recently been recognized as a driving force in the organization of cellular content. Many of the bona fide condensates that have been studied in cells or in the test tube contain DNA or RNA. Examples are heterochromatin compartments, nucleoli, or nuclear bodies, whose distinct physicochemical properties are believed to be linked to their biological function (27,28). These properties will depend both on the protein and the nucleic acid components, which has, for example, been documented for model protein-RNA condensates (29). Likewise, condensates that reside in the nucleus and contain genomic DNA will be influenced by the phase behavior of the enclosed DNA. Although it has been shown that the latter depends on the DNA sequence and flexibility (30, 31, 32), the relevance of other DNA features has not extensively been assessed. In particular, it is currently unclear how strongly the length of the enclosed DNA affects the material properties of the resulting condensate.

Here, we addressed this issue and compared condensates of DNA molecules with different lengths formed via crowding polymers and salt, binding of linker histone H1, or nucleosome reconstitution in combination with linker histone H1. The first type of condensate does not contain any protein component and therefore reflects the length-dependent phase behavior of DNA itself. The other types of condensates serve as simplified versions of the scenario in the cell, in which DNA interacts with positively charged histones. We find that short DNA molecules can form liquid-like condensates under all sets of conditions tested here, suggesting that DNA has the intrinsic propensity to undergo liquid-liquid phase separation, also in the absence of any disordered proteins. This finding is consistent with recent work that has reported liquid-like DNA condensates under various conditions (11,18,19,31,33, 34, 35). However, we preferentially observed liquid-like condensates with short DNA molecules, which are much smaller than a mammalian chromosome or even a single gene. Longer DNA molecules, e.g., phage *λ*-DNA with a size of 48.5 kb, preferentially formed solid-like structures. It is conceivable that the basis for this length dependence is that short DNA fragments interact transiently with one another, whereas interactions add up for longer DNA molecules to favor the transition to a more solid-like or gel-like state, in which the mobility of DNA molecules is restricted due to the increased number of interactions per molecule. Such behavior is compatible with a polymer-polymer phase separation process, which drives the coalescence of segments of long polymers into a reordered state (28,36,37). A conceptually similar relationship between fluidity and polymer length has recently been described for repeat-containing RNAs (38) and proteins (39,40), suggesting that this phenomenon is a fundamental biophysical property of cellular macromolecules.

## Conclusions

**Figure 7:**
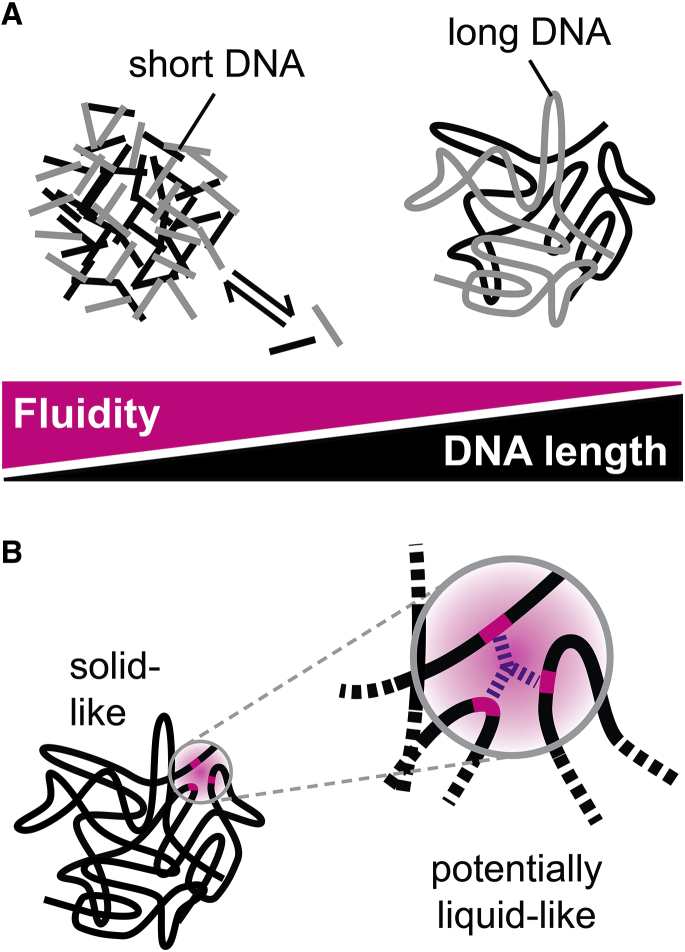
Length-dependent fluidity of DNA condensates. (*A*) DNA length and fluidity of condensates were anticorrelated for the condensation conditions we used here. (*B*) Although long DNA molecules as a whole tend to form solid-like condensates, liquid-like properties might be observed on small scales if only short parts (*magenta*) of a long DNA molecule (*black*) are engaged in attractive interactions. To see this figure in color, go online.

In this study, we systematically assessed the material properties of DNA condensates formed with differently sized DNA molecules and established a link between DNA length and fluidity. Our results suggest that entire chromosomes or large chromosomal domains such as pericentric heterochromatin tend to condense into rather solid-like structures (Fig. 7 *A*), which is consistent with the morphology and dynamics of such chromosomal domains as reviewed recently (36). In contrast, liquid-like condensates are expected to be preferentially obtained with relatively short DNA molecules in the test tube. This length dependence should be taken into account when extrapolating from the behavior of in vitro condensates to that of cellular structures containing large genomic regions. Our results allow us to estimate the length scale below which short segments of different DNA molecules or of the same DNA molecule might coalesce into liquid-like condensates. For the three conditions we tested here, this length scale turned out to be around 1–10 kb, suggesting that small genomic regions with the size of promoters/enhancers might be able to form liquid-like condensates if they engage in attractive interactions and the intervening regions do not (Fig. 7 *B*). We would like to point out that the length dependence documented here represents the inherent phase behavior of DNA, which will likely be modulated in the cell by a plethora of chromatin-associated factors and active processes that act on the DNA. Nevertheless, we expect that the general trend will be preserved and that cellular processes fine-tune the length-dependent fluidity of DNA-based condensates without entirely overriding their inherent behavior.

Taken together, we show here that DNA has the intrinsic capacity to condense into liquid-like or solid-like assemblies and that the properties of DNA condensates critically depend on DNA length. We anticipate that our work will help reconcile conflicting observations of DNA-containing condensates made in vitro and in cells and will motivate experimental designs that account for the impact of DNA length on cellular phase separation processes.

## Author contributions

Acquisition of data: F.M. and M.H. Analysis of data: F.M., M.H., and F.E. Drafting of manuscript: F.M. and F.E. Manuscript reviewing: F.M., M.H., and F.E. Supervision, study design, and coordination: F.E.

## Supporting material

Document S1. Figs. S1–S7 and Tables S1–S4

Data S1. Calculation of the expansion parameter that minimizes the free energy

Data S2. Calculation of phase diagrams

Figure360. An Author Presentation of Figure 6

Document S2. Article plus supporting material
