# Supplementary material for: DNA length tunes the fluidity of DNA-based condensates
Source: Biophys J. 2021 Feb 26;120(7):1288–300. doi: 10.1016/j.bpj.2021.02.027 (PMC8059207; doi:10.1016/j.bpj.2021.02.027)
Supplement: Document S1. Figs. S1–S7 and Tables S1–S4 [file mmc1.pdf]

Biophysical Journal, Volume *120*

Supplemental information

DNA length tunes the fluidity of DNA-based condensates

Fernando Muzzopappa, Maud Hertzog, and Fabian Erdel

Supporting Material for "DNA length tunes the fluidity of DNA-based condensates"
by F. Muzzopappa, M. Hertzog, F. Erdel

Content

Supporting Figures

Figure S1. Size distribution of DNA samples and characterization of reconstituted chromatin.
Figure S2. Aspect ratio and size of DNA condensates.
Figure S3. Morphology and behavior of DNA condensates.
Figure S4. Partial FRAP of DNA condensates.
Figure S5. Effect of λ-DNA addition to condensates containing short DNA.
Figure S6. Differential dynamics in H1- and PSI-condensates.
Figure S7. Stability of DNA condensates in flow.

Supporting Tables

Table S1. List of oligonucleotides.
Table S2. Aspect ratios of DNA condensates.
Table S3. FRAP fit results.
Table S4. Condensate stability fit results.

Supporting Figures

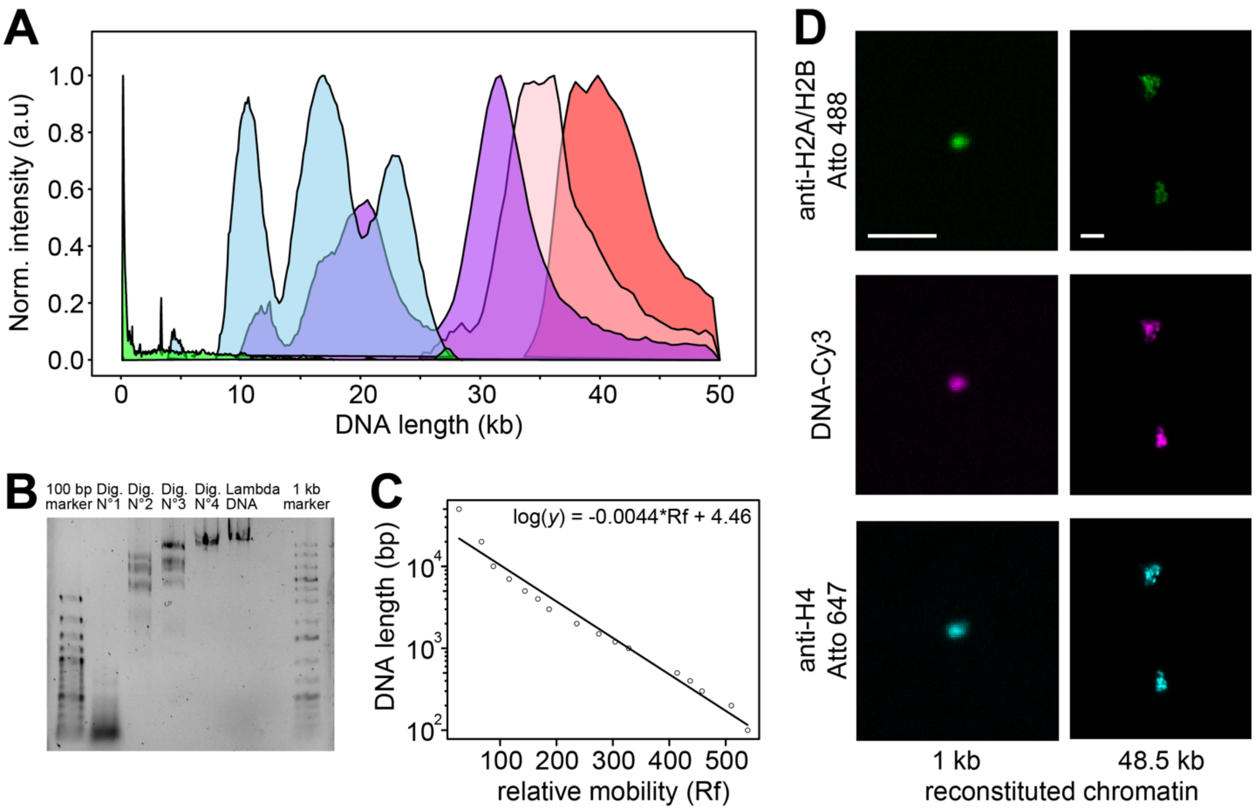

Figure S1. Size distribution of DNA samples and characterization of reconstituted chromatin.

(A) Migration profile of λ-DNA (red), AfeI-digested λ-DNA (pink), BamHI/KpnI-digested λ-DNA (purple), BamHI/KpnI/AatII-digested λ-DNA (skyblue) and AciI/HindIII/DraI-digested λ-DNA (green), obtained from the 0.8% agarose gel depicted in panel B. The length of the DNA fragments (in kb) was calculated from the calibration curve in panel C.

(B) Agarose gel showing the different digested λ-DNA species used in this study together with the DNA ladders 1 kb Plus (Thermo, SM1333) and 100 bp Plus (Thermo, SM0323).

(C) Calibration curve based on the gel in panel B.

(D) Representative images of condensates formed with reconstituted chromatin and linker histone H1. The presence of histones H2A/H2B and H4 was verified via immunostaining.

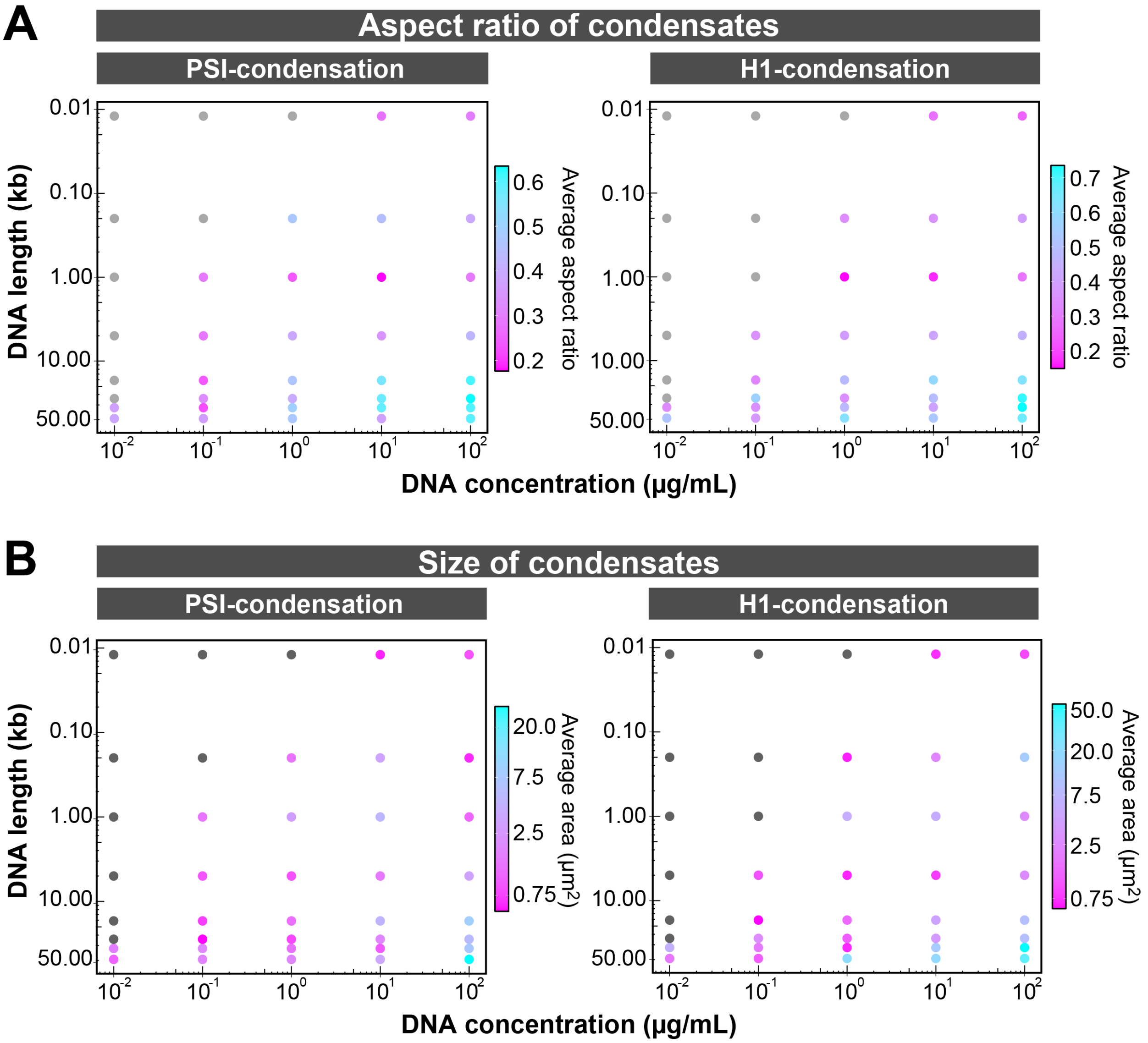

Figure S2. Aspect ratio and size of DNA condensates.

(A) Phase diagrams of PSI DNA condensates (left) and H1-induced condensates (right). Colors represent the average aspect ratio of the condensates. Gray points indicate conditions in which no DNA condensation was observed.

(B) Same as panel A but for the average area of the condensates.

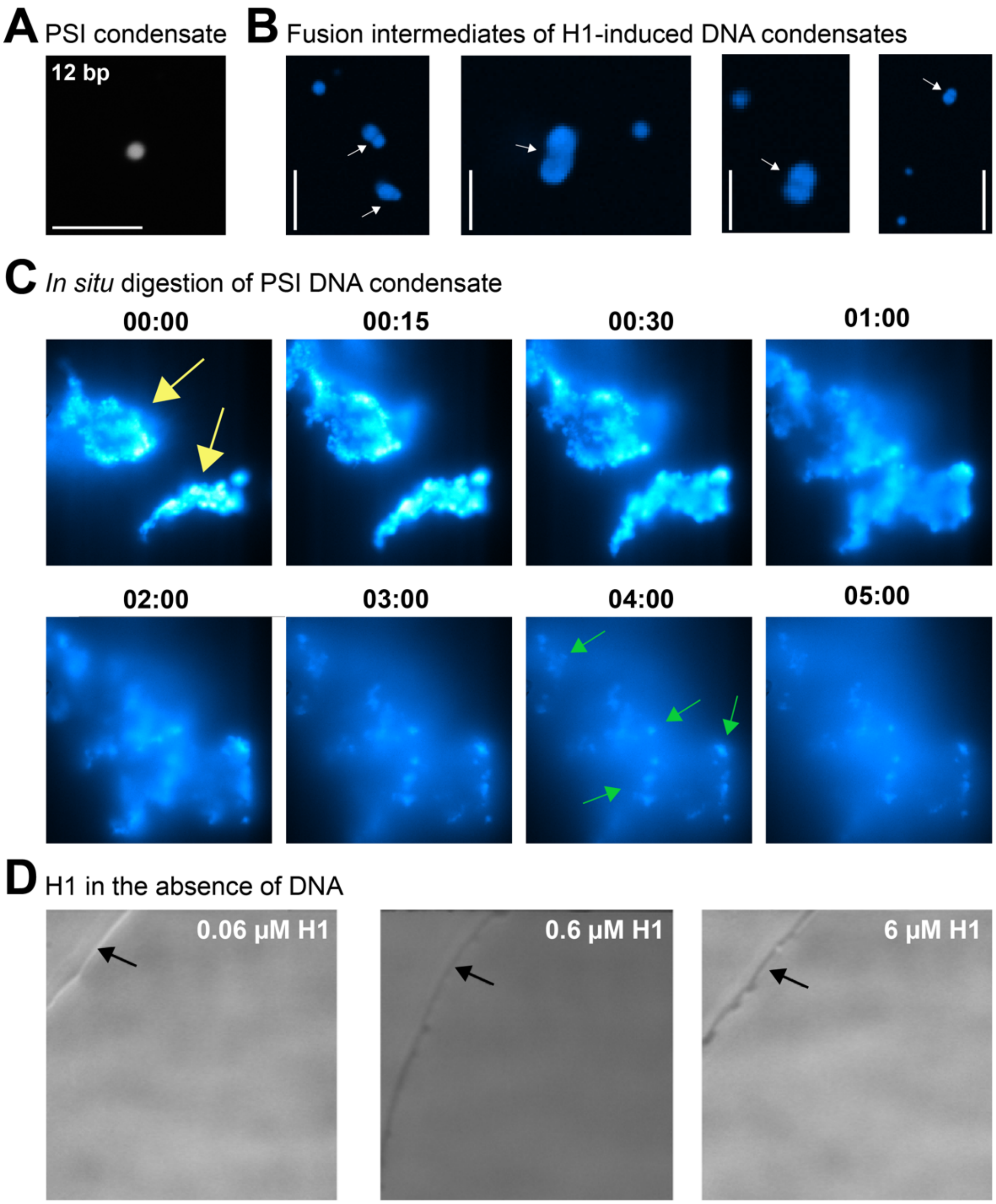

Figure S3. Morphology and behavior of DNA condensates.

(A) Representative image of a round-shaped 12 bp PSI DNA condensate. Scale bar, 5 µm.

(B) Representative images of *bona fide* intermediates of fusion events between condensates containing 12 bp-DNA molecules that were formed via H1-induced condensation. Arrows indicate the intermediates. Scale bars, 5 µm.

(C) Time-lapse of *in situ* digestion of an irregularly-shaped DNA condensate performed by addition of micrococcal nuclease to DNA (digested λ-DNA, 28 kb). Images were acquired for 5 minutes. Yellow arrows mark the initial irregular condensates and green arrows mark the resulting smaller condensates.

(D) Bright-field images of H1 alone (without DNA) at the working concentrations used in the experiments. No H1 condensates were observed under these conditions. The arrows mark the air-liquid interphase.

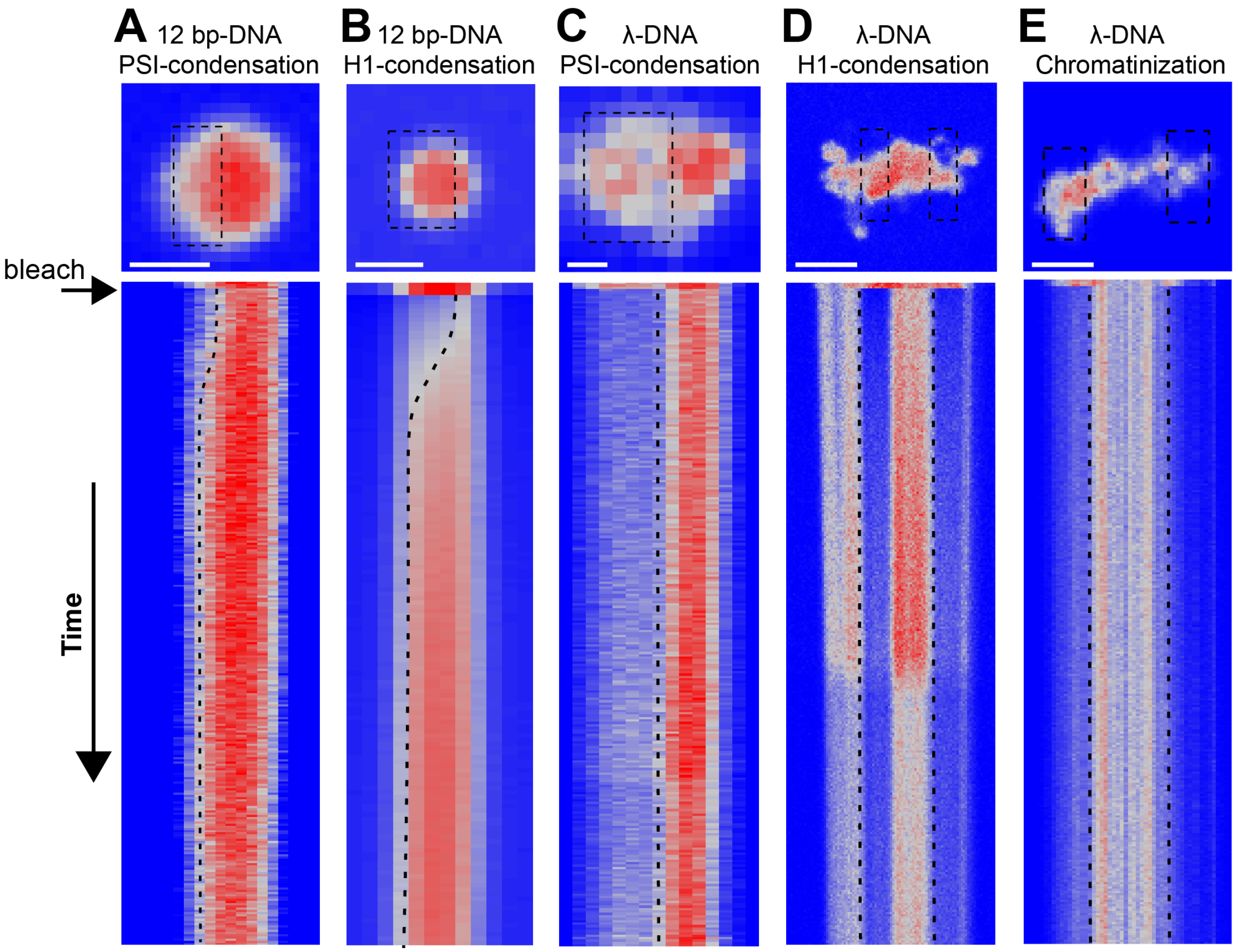

Figure S4. Partial FRAP of DNA condensates.
Partial photobleaching of condensates containing 12 bp-DNA (panels A and B) or λ-DNA (panels C-E) induced by PSI-condensation, H1-condensation or chromatinization as indicated. Pre-bleach images are shown in the top panels, with dashed rectangles indicating the bleach regions. Bottom panels show kymographs of the fluorescence recovery. Scale bars, 1 μm (panels A and B) or 5 μm (panels C-E).

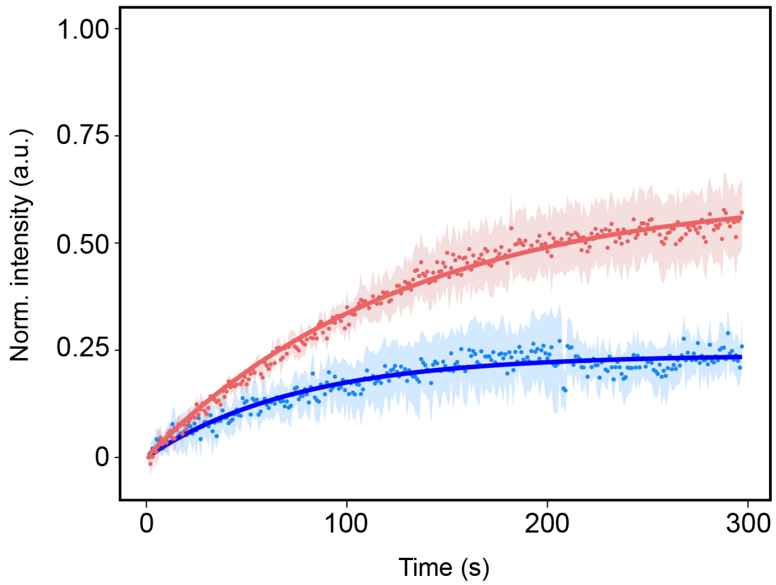

Figure S5. Effect of λ-DNA addition to condensates containing short DNA.
FRAP recovery curves for DNA condensates formed with 12 bp-DNA at 100 µg/mL (red) and 12 bp-DNA at 100 µg/mL in the presence of the same mass of λ-DNA (blue). Points correspond to experimental data, lines represent mono-exponential fits. Error bars (shaded areas) represent the SEM.

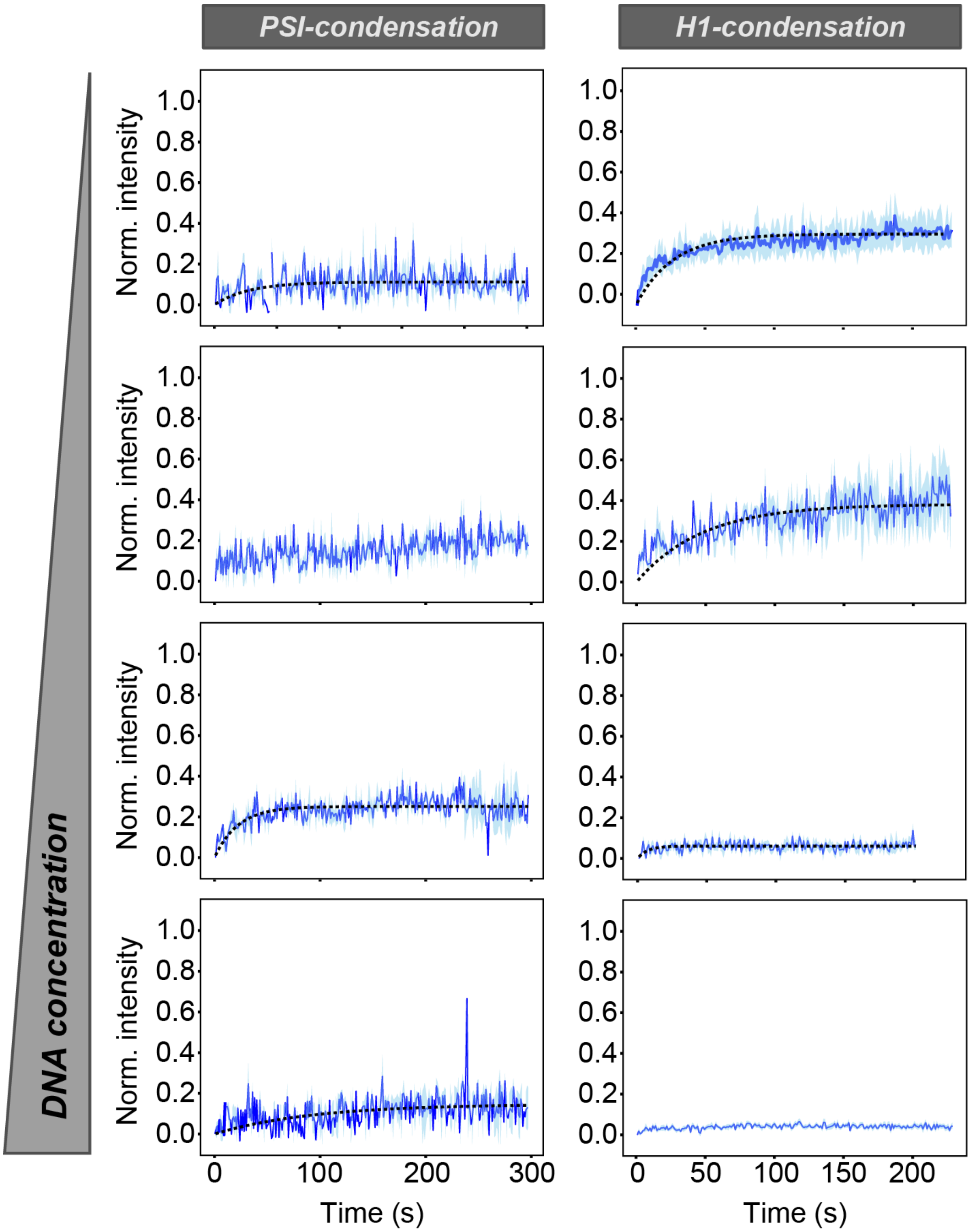

Figure S6. Differential dynamics in H1- and PSI-condensates.

FRAP recovery curves for DNA condensates containing 28 kb-DNA molecules. Left panels correspond to PSI-condensates and right panels to H1-induced condensates. From top to bottom, the following DNA concentrations were used: 0.1 µg/mL, 1 µg/mL, 10 µg/mL and 100 µg/mL. Error bars (shaded areas) represent the SEM.

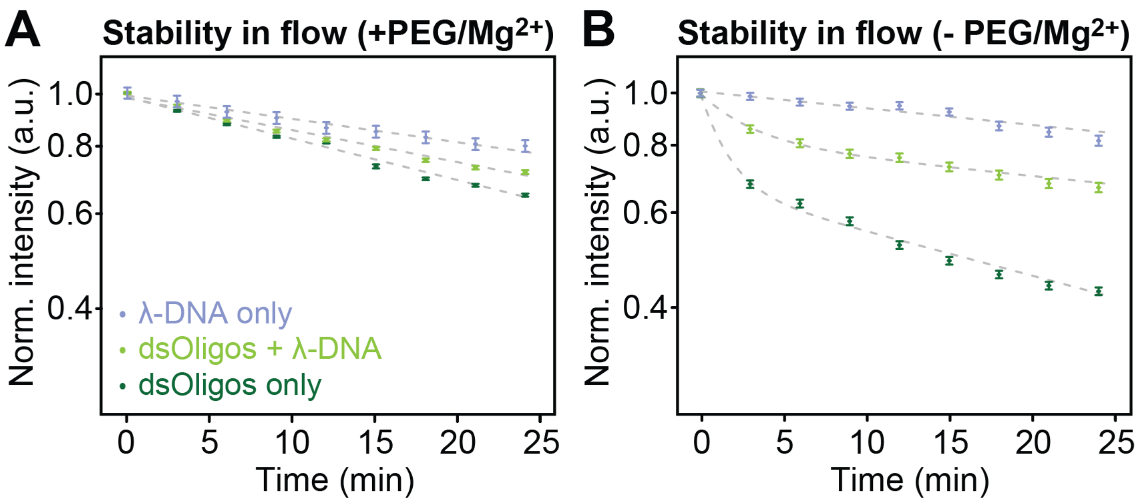

Figure S7. Stability of DNA condensates in flow.

(A) Stability of DNA condensates in PSI condensation buffer under flow. The signal in the red channel is shown. Error bars, SEM.

(B) Same as panel A but for decondensation buffer.

Supporting Tables

Name	Sequence	Modifications
Lambda_R	AGGTCGCCGCCC	Cy3 (5')
Lambda_L	GGGCGGCGACCT	Cy3 (5')
LamdaFW	CGGGTTTTCGCTATTTATGAAAATTTTCCG	-
Lambda1_rv	CAGATAAGGGTGTTGCGCTGC	-
Lambda5_rv	CTGTGAGCCATCATGACGCCGATGG	-
LamdaRV	CATCTACATATCACAACGTGCGTGGAGG	-
Lambda1_fw	GCCATTGGTAAAACCTTCCATGTGATACG	-
Lambda5_fw	ATCAGAGCGTGGAAACGTTAATCACTGG	-
pUC_Bio_fw	CTCGGTACCCGGGGATCCTCTAGAG	Biotin (5')
pUC_fw	CTCGGTACCCGGGGATCCTCTAGAG	-
pUC_A488_rv	AGAGGATCCCCGGGTACCGAGTCGTCG	Atto 488 (5')
pUC_A565_rv	AGAGGATCCCCGGGTACCGAGTCGTCG	Atto 565 (5')

Table S1. List of oligonucleotides.
The sequences and chemical modifications of all oligonucleotides used in this study are listed.

Aspect ratio

PSI-condensation

c (mg/L)	48.5 kb	36 kb	28 kb	17 kb	5 kb	1 kb	0.2 kb	0.012 kb
100	0.59 ± 0.20	0.60 ± 0.21	0.63 ± 0.20	0.61 ± 0.23	0.43 ± 0.18	0.29 ± 0.13	0.39 ± 0.16	0.30 ± 0.13
10	0.38 ± 0.10	0.59 ± 0.17	0.58 ± 0.22	0.56 ± 0.19	0.35 ± 0.11	0.18 ± 0.07	0.45 ± 0.12	0.28 ± 0.10
1	0.47 ± 0.21	0.49 ± 0.19	0.40 ± 0.16	0.46 ± 0.19	0.39 ± 0.22	0.24 ± 0.11	0.36 ± 0.12	
0.1	0.37 ± 0.11	0.22 ± 0.03	0.33 ± 0.20	0.24 ± 0.08	0.28 ± 0.11	0.29 ± 0.11		
0.01	0.39 ± 0.11	0.37 ± 0.22						

H1-condensation

c (mg/L)	48.5 kb	36 kb	28 kb	17 kb	5 kb	1 kb	0.2 kb	0.012 kb
100	0.66 ± 0.16	0.73 ± 0.21	0.72 ± 0.18	0.63 ± 0.22	0.46 ± 0.15	0.28 ± 0.14	0.39 ± 0.11	0.25 ± 0.13
10	0.53 ± 0.14	0.41 ± 0.17	0.49 ± 0.17	0.59 ± 0.18	0.42 ± 0.15	0.17 ± 0.06	0.36 ± 0.22	0.28 ± 0.12
1	0.62 ± 0.21	0.48 ± 0.20	0.36 ± 0.11	0.49 ± 0.21	0.39 ± 0.13	0.15 ± 0.04	0.35 ± 0.09	
0.1	0.39 ± 0.15	0.35 ± 0.05	0.57 ± 0.28	0.33 ± 0.12	0.36 ± 0.09			
0.01	0.51 ± 0.16	0.34 ± 0.04						

Chromatinization (reconstituted chromatin with H1)

c (mg/L)	48.5 kb	36 kb	28 kb	17 kb	5 kb	1 kb
1	0.67 ± 0.21	0.70 ± 0.16	0.57 ± 0.14	0.51 ± 0.18	0.47 ± 0.17	0.46 ± 0.17

Number of analyzed condensates

PSI-condensation

c (mg/L)	48.5 kb	36 kb	28 kb	17 kb	5 kb	1 kb	0.2 kb	0.012 kb
100	16	29	42	42	33	385	94	414
10	28	27	17	14	44	18	66	213
1	22	25	12	13	31	25	41	
0.1	13	8	11	13	10	12		
0.01	11	13						

H1-condensation

c (mg/L)	48.5 kb	36 kb	28 kb	17 kb	5 kb	1 kb	0.2 kb	0.012 kb
100	18	10	21	12	52	321	11	91
10	12	11	31	22	145	32	25	40
1	15	71	41	30	15	26	9	
0.1	67	22	13	21	11			
0.01	12	10						

Chromatinization (reconstituted chromatin with H1)

c (mg/L)	48.5 kb	36 kb	28 kb	17 kb	5 kb	1 kb
1	10	15	23	11	74	336

Table S2. Aspect ratios of DNA condensates.

Aspect ratios measured for DNA condensates under different conditions. Errors represent the standard deviation. The number of DNA condensates analyzed for each condition is indicated in the bottom half of the table.

FRAP results for PSI DNA condensates

**DNA size**	12 bp + 48.5 kb	12 bp	12 bp	200 bp	200 bp	200 bp
**DNA conc. (µg/mL)**	100	100	10	100	10	1
**k (min^{-1})**	0.79 ± 0.03	0.42 ± 0.01	1.6 ± 0.1	2.6 ± 0.1	1.5 ± 0.1	2.5 ± 0.1
**$t_{1/2}$ (s)**	53	99	27	16	29	17
**Mobile fraction (%)**	23 ± 1	66 ± 1	74 ± 1	72 ± 1	82 ± 1	47 ± 1

FRAP results for PSI DNA condensates

**DNA size**	17 kb	28 kb	28 kb	28 kb	36 kb	48.5 kb
**DNA conc. (µg/mL)**	100	10	1	0.1	1	1
**k (min^{-1})**	1.3 ± 0.5	2.7 ± 0.2	3.0 ± 0.7	2.2 ± 0.5	0.7 ± 0.1	*2.6 ± 0.8*
**$t_{1/2}$ (s)**	33	16	14	20	63	*16*
**Mobile fraction (%)**	16 ± 1	25 ± 1	11 ± 1	11 ± 1	14 ± 1	8 ± 1

FRAP results for H1-induced DNA condensates

**DNA size**	12 bp	12 bp	200 bp	200 bp	200 bp	17 kb
**DNA conc. (µg/mL)**	100	10	100	10	1	100
**k (min^{-1})**	0.70 ± 0.02	0.45 ± 0.02	0.30 ± 0.03	4.2 ± 0.6	0.9 ± 0.1	0.4 ± 0.6
**$t_{1/2}$ (s)**	60	94	140	11	46	99
**Mobile fraction (%)**	58 ± 1	62 ± 1	72 ± 4	46 ± 2	45 ± 1	15 ± 1

FRAP results for H1-induced DNA condensates

**DNA size**	28 kb	28 kb	28 kb	36 kb	36 kb	48.5 kb
**DNA conc. (µg/mL)**	10	1	0.1	1	0.01	1
**k (min^{-1})**	*8 ± 2*	1.3 ± 0.1	2.3 ± 0.1	11 ± 3	2.7 ± 0.2	1.1 ± 0.1
**$t_{1/2}$ (s)**	*5*	33	19	4	15	37
**Mobile fraction (%)**	6 ± 1	38 ± 1	35 ± 1	10 ± 1	35 ± 1	28 ± 1

FRAP results for reconstituted chromatin with H1

**DNA size**	1 kb	5 kb	17 kb	28 kb	36 kb	48.5 kb
**DNA conc. (µg/mL)**	1	1	1	1	1	1
**k (min^{-1})**	0.60 ± 0.32	0.67 ± 0.04	0.35 ± 0.02	*0.86 ± 0.27*	*2.10 ± 0.06*	*0.84 ± 0.03*
**$t_{1/2}$ (s)**	69	62	120	*48*	*20*	*50*
**Mobile fraction (%)**	40 ± 1	15 ± 1	19 ± 1	9 ± 1	10 ± 1	8 ± 1

Table S3. FRAP fit results.
Fit results for different FRAP experiments using the equation given in the Materials and Methods section, with k being the apparent exchange rate and $t_{1/2}$ the half-recovery time. At least five curves were averaged for each condition. Rates and half-recovery times written in blue italics correspond to FRAP curves with mobile fractions ≤10%, indicating that the respective condensates were mostly immobile and that these parameters only refer to a small subset of molecules in the condensate. Errors represent standard fit errors.

PSI condensation buffer						
**Condition**	dsOligos only, green	dsOligos only, red	dsOligos + λ-DNA, green	dsOligos + λ-DNA, red	λ-DNA, green	λ-DNA, red
***k* (min^{-1})**	< 0.001	0.018 ± 0.001	0.004 ± 0.001	0.014 ± 0.001	0.008 ± 0.001	0.010 ± 0.001
Decondensation buffer						
**Condition**	dsOligos only, green	dsOligos only, red	dsOligos + λ-DNA, green	dsOligos + λ-DNA, red	λ-DNA, green	λ-DNA, red
**k_{fast} (min^{-1})**	0.46 ± 0.08	0.62 ± 0.17	0.21 ± 0.08	0.33 ± 0.11	-	-
**k_{slow} (min^{-1})**	0.019 ± 0.004	0.019 ± 0.002	0.003 ± 0.005	0.007 ± 0.002	0.004 ± 0.002	0.005 ± 0.002
**f_{fast} (%)**	55 ± 4	33 ± 3	32 ± 8	19 ± 3	-	-

Table S4. Condensate stability fit results.
Fit results for single-condensate stability experiments shown in ***Figs. 4*** and ***S7***. For dsOligos only, 975 condensates were analyzed; for dsOligos + λ-DNA, 770 condensates were analyzed; for λ-DNA only, 284 condensates were analyzed. Red and green signals were acquired in the same experiments. Curves for PSI condensation buffer were fitted with a mono-exponential decay (rate k), curves for decondensation buffer were fitted with a double-exponential decay (rates k_{fast} and k_{slow}, fast and slow fractions f_{fast} and $1-f_{fast}$). The slow decay rates likely correspond to photobleaching. Errors represent standard fit errors.
